# WNK3 inhibition elicits antitumor immunity by suppressing PD-L1 expression on tumor cells and activating T-cell function

**DOI:** 10.1038/s12276-022-00876-z

**Published:** 2022-11-10

**Authors:** Hyun Ju Yoon, Gi-Cheon Kim, Sejin Oh, Hakhyun Kim, Yong Keon Kim, Yunji Lee, Min Seo Kim, Gino Kwon, Yeon-Su Ok, Ho-Keun Kwon, Hyun Seok Kim

**Affiliations:** 1 Severance Biomedical Science Institute, Yonsei University College of Medicine, Seoul, Korea; 2 Graduate School of Medical Science, Brain Korea 21 Project, Yonsei University College of Medicine, Seoul, Korea; 3 Department of Microbiology and Immunology, Yonsei University College of Medicine, Seoul, Korea; 4 Institute for Immunology and Immunological Diseases, Yonsei University College of Medicine, Seoul, Korea; 5 Graduate Program for Nanomedical Science, Yonsei University, Seoul, Korea

**Keywords:** Cancer immunotherapy, Non-small-cell lung cancer, RNAi

## Abstract

Immune checkpoint therapies, such as programmed cell death ligand 1 (PD-L1) blockade, have shown remarkable clinical benefit in many cancers by restoring the function of exhausted T cells. Hence, the identification of novel PD-L1 regulators and the development of their inhibition strategies have significant therapeutic advantages. Here, we conducted pooled shRNA screening to identify regulators of membrane PD-L1 levels in lung cancer cells targeting druggable genes and cancer drivers. We identified WNK lysine deficient protein kinase 3 (WNK3) as a novel positive regulator of PD-L1 expression. The kinase-dead WNK3 mutant failed to elevate PD-L1 levels, indicating the involvement of its kinase domain in this function. WNK3 perturbation increased cancer cell death in cancer cell–immune cell coculture conditions and boosted the secretion of cytokines and cytolytic enzymes, promoting antitumor activities in CD4^+^ and CD8^+^ T cells. WNK463, a pan-WNK inhibitor, enhanced CD8^+^ T-cell-mediated antitumor activity and suppressed tumor growth as a monotherapy as well as in combination with a low-dose anti-PD-1 antibody in the MC38 syngeneic mouse model. Furthermore, we demonstrated that the c-JUN N-terminal kinase (JNK)/c-JUN pathway underlies WNK3-mediated transcriptional regulation of PD-L1. Our findings highlight that WNK3 inhibition might serve as a potential therapeutic strategy for cancer immunotherapy through its concurrent impact on cancer cells and immune cells.

## Cancer treatment: A new approach to boosting anti-tumor immunity

Inhibiting the activity of the protein WNK lysine deficient protein kinase 3 (WNK3) boosts anti-tumor immunity by suppressing the production of another protein (PD-L1) whose activity promotes the programmed cell death of immune system T cells. Hyun Seok Kim, Ho-Keun Kwon and colleagues at Yonsei University in Seoul, South Korea, undertook a wide search for factors that could regulate PD-L1 levels in human lung cancer cells. While identifying WNK3 inhibition as a route towards restoring the anti-cancer activity of T cells, the research also revealed details of the molecular signaling pathways that allow WNK3 to affect PD-L1 production. Tests in mice confirmed that WNK3 inhibition can suppress tumor growth. Drugs that inhibit WNK3 can now be investigated as a new approach to treating cancer, due to their effects on both immune system cells and cancer cells.

## Introduction

The immune system recognizes and eradicates malignant neoplasms. However, genetic alterations acquired during the evolution of tumor cells enable them to evade immune surveillance^1^. PD-L1, expressed on either cancer cells or cancer-associated immune cells, binds to its respective immune checkpoint receptor, programmed cell death 1 (PD-1), which induces an inhibitory signal in activated T cells to promote T-cell apoptosis, anergy, and functional exhaustion^2^. Therefore, blocking the PD-L1/PD-1 axis restores T-cell function and induces durable tumor remission in cancer patients, especially those with melanoma, non-small-cell lung cancer, renal cell carcinoma, and others^3^. Current therapeutic strategies for blocking the PD-L1/PD-1 axis use various monoclonal antibodies against PD-L1 or PD-1. However, in-depth investigations are required to gain adequate knowledge of the genetic regulators underlying tumor-specific PD-L1 upregulation. In particular, identifying novel regulators and developing strategies to inhibit the PD-L1/PD-1 axis may provide additional avenues for advancing immune checkpoint therapies.

Interferon-γ (IFN-γ), the inflammatory cytokine released by infiltrated T cells, is the most well-known factor that induces PD-L1 in the tumor microenvironment. IFN-γ binds to its cognate receptor and stimulates PD-L1 expression by activating the Janus kinase (JAK)/signal transducer and activator of transcription (STAT) pathway^4^. Moreover, intrinsic oncogenic signaling enhances PD-L1 expression to protect cancer cells from immune cell attack. Oncogenes such as epidermal growth factor receptor (EGFR), ALK receptor tyrosine kinase (ALK), mitogen-activated protein kinase (MAPK), MET proto-oncogene (MET), AKT serine/threonine kinase (AKT), MYC proto-oncogene (MYC), STAT3, cyclin dependent kinase 5 (CDK5), and Yes associated transcriptional regulator (YAP) enhance PD-L1 expression. Recently, genome-wide screenings were performed for systematic identification of PD-L1 regulators. Two studies using CRISPR‒Cas9-mediated loss-of-function screening in pancreatic cancer cells^5^ and haploid genetic screening in HAP1 cells^6^ identified CKLF-like MARVEL transmembrane domain containing 6 (CMTM6) as a PD-L1 enhancer. More recently, CRISPR‒Cas9-based screenings in lung and colon cancer cell lines identified eukaryotic translation initiation factor 5B (eIF5B) and myeloid/lymphoid or mixed-lineage leukemia, translocated to 6 (MLLT6) as novel regulators of PD-L1 abundance, respectively^7,8^. These unbiased, systematic approaches highlighted the power of functional genomics-based screening methods for identifying novel PD-L1 regulators.

However, the pharmacological intractability of the identified genes limits the translational implications of these studies. Hence, we assembled a focused shRNA library that specifically targets druggable genes and cancer drivers in the genome, allowing higher in-depth phenotypic analysis of each shRNA target than whole genome screening on the same scale. We hypothesized that this focused functional genomic screening of druggable genes and cancer drivers can evaluate the impact of perturbing such genes on immune evasion, particularly through PD-L1 regulation. Hence, we set up a phenotypic screening system to identify enhancers and suppressors of PD-L1 membrane expression with custom-made shRNA pools.

## Materials and methods

### Cell lines

Human NSCLC cell lines (H2009, H460, HCC44, HCC461, H647, H2122, and A549) were kindly provided by Michael A. White and John D. Minna (UT Southwestern Medical Center, TX, USA). The cell lines were maintained in RPMI-1640 medium supplemented with 5% fetal bovine serum (Gibco/Thermo Fisher Scientific, Waltham, MA, USA) and 1% penicillin‒streptomycin (Invitrogen, Carlsbad, CA, USA). The Jurkat T-cell line was purchased from ATCC (Manassas, VA, USA) and was maintained in RPMI-1640 medium supplemented with 10% fetal bovine serum and 1% penicillin‒streptomycin. The mouse MC38 cell line was purchased from Kerafast (Boston, MA, USA). The MC38 cell line was cultured in Dulbecco’s modified Eagle’s medium (DMEM) (Gibco/Thermo Fisher Scientific) supplemented with 10% fetal bovine serum and 1% penicillin‒streptomycin.

### Construction of pooled shRNA libraries

Lists of druggable genes and cancer drivers were obtained from the drug-gene interaction database^9^ (Supplementary Table 1) and pan-cancer genome atlas studies^10–17^ (Supplementary Table 2), respectively, which correspond to 32,643 shRNAs for 5069 druggable genes and 5611 shRNAs for 800 cancer driver genes. These were combined into a single library containing 35,949 shRNAs for 5592 genes.

For preparation of the shRNA pools, bacterial stocks were cherry-picked from the MISSION shRNA library (Sigma‒Aldrich, St. Louis, MO, USA) and plated on LB agar plates using a BioMek FXII liquid handler (Beckman Coulter, Brea, CA, USA). After overnight incubation at 37 °C, bacterial clones were pooled, and DNA was purified with a DNA-mag Plasmid DNA purification kit (#17255, iNtRON Biotechnology, Seongnam-si, Korea). HEK293FT cells (1 × 10^7^) were allowed to adhere to 150-mm dishes overnight. Lentivirus was produced by cotransfecting HEK293FT cells with 7.2 μg of the plasmid DNA pool, 5.4 μg of psPAX2 and 1.8 μg of pMD2.G (Addgene, Watertown, MA, USA). The culture medium was changed 24 h after transfection, and the viral supernatant was collected twice at 48 h and 72 h after transfection and pooled. The lentivirus titer was determined using an HIV Type 1 p24 Antigen ELISA kit (#0801111, Zeptometrix, City Road, London, UK) in accordance with the manufacturer’s protocol.

### Lentiviral production and shRNA screen

H2009 cells (4.8 × 10^7^) were transduced with lentivirus carrying pooled shRNA libraries at an MOI of 0.3 and selected with 1 μg/ml puromycin for 72 h, starting 48 h after transduction. Selected cells were sorted based on PD-L1 expression levels on the cell surface with BD FACS Aria III (BD Biosciences, Franklin Lakes, NJ, USA) at Day 3 [t0], Day 13 [t1], and Day 20 [t2]. For sorting, 1.8 × 10^7^ cells were collected using 10 mM EDTA, stained with 5 μg/ml PE anti-human PD-L1 (#12-5983-42, eBioscience, San Diego, CA, USA) in PBS containing 0.5% BSA for 30 min on ice and washed with PBS containing 0.5% BSA. The PD-L1-low population was gated with reference to unstained control cells, and PD-L1-high and PD-L1-intermediate populations were 1–2% and 13–15% of the total cells with high and intermediate PD-L1 expression, respectively. The unsorted pool of 1.8 × 10^7^ cells was harvested at [t0] and [t2], and the remaining cells were subcultured for the next round of FACS sorting.

Genomic DNA was isolated from FACS-sorted and unsorted cells using a QIAamp DNA Blood Mini Kit (#51104, QIAGEN, Germantown, MD, USA). shRNA inserts were amplified with two rounds of PCR (20 cycles per round) using GoTaq Hot Start Polymerase (#M5001, Promega, Madison, WI, USA). The annealing temperatures for the first and second rounds of PCR were set to 52 °C and 56 °C, respectively. Primer sequences for the first round of PCR were as reported by Cowley GS et al.^18^. The second-round PCR primers were custom-designed to have Illumina P5 and P7 adapters and to contain variable sequences with stagger regions to maintain base diversity in the first 16 cycles of the sequencing process, during which clusters are called. In addition, by using barcoded reverse primers, all samples were multiplexed in a single lane. The primer sequences are provided in Supplementary Table 5. The PCR products purified using the AMPure XP purification system (#A63880, Beckman Coulter) were sequenced with paired-end 100-bp reads on a HiSeq2000, achieving an average coverage of 1000x of the shRNA pools.

Both sense sequences and antisense sequences of shRNAs were extracted by removing the remaining sequences of raw reads using Cutadapt^19^. Sequences were aligned to the reference shRNA sequences with BWA, allowing up to 3-nt mismatches, and aligned reads were counted with Bedtools^20^. Only the reads with concordant mapping to a single shRNA sequence both by sense and antisense sequences were subjected to counting, and reads whose sum of counts in PD-L1-low, PD-L1-intermediate, and PD-L1-high populations was less than 100 were excluded. Read counts were normalized to the total number of reads and the number of cells in each sample. Then, the PD-L1-low population enrichment score was calculated with the equation log2 low/(intermediate+high), where low, intermediate, and high are the normalized read counts extracted from samples with low, intermediate, and high PD-L1 expression. The PD-L1-high population enrichment score was calculated in the same manner. The enrichment scores were analyzed using RIGER, the second-best algorithm^21^, to convert the shRNA-level scores into gene-level scores and rank the genes for which targeting shRNA was highly enriched in the sorted cell populations. This ranking algorithm computes gene scores based on the second-best scoring shRNA for that gene.

### T-cell-mediated tumor cell killing assay

Tumor cells (6–7 × 10^3^) were allowed to adhere to 96-well plates overnight and then were incubated with human peripheral blood mononuclear cells (PBMCs; #70025, Stemcell Technologies, Vancouver, BC, Canada) to produce different target-to-effector ratios (1:5, 1:10). To activate T cells, 100 ng/ml anti-CD3 antibody and 10 ng/ml IL-2 (#317325, #589102, BioLegend, San Diego, CA, USA) were added to the wells. After 48–96 h of coculture, PBMCs were removed and washed with PBS, and cancer cell viability was measured with the CellTiter-Glo assay kit (Promega). PBMC-mediated cancer cell death was assessed by normalizing the cancer cell viability of the test wells (1:5, 1:10) to that of the control wells without PBMCs.

### Cytokine assays

PBMCs (2 × 10^5^) activated with 100 ng/ml anti-CD3 antibody and 10 ng/ml IL-2 or with Dynabeads Human T-Activator CD3/CD28 (#11161D, Gibco) at a bead-to-cell ratio of 10:1 were incubated with the test compounds for 3 days. Granzyme B levels in the collected supernatants were determined using a LEGEND MAX™ Human Granzyme B ELISA Kit (#439207, BioLegend) in accordance with the manufacturer’s protocol. Jurkat T cells (5 × 10^4^) were activated by Dynabeads Human T-Activator CD3/CD28 (#11161D, Gibco) at different bead-to-cell ratios (0:1, 5:1, and 10:1) for 48 h. The IL-2 concentration in the supernatants was measured by a LEGEND MAX™ Human IL-2 ELISA Kit (#431807, BioLegend) following the manufacturer’s protocol. The levels of 12 molecular species in the culture supernatant were quantified using a Luminex platform (Human Th Cytokine Panel, BioLegend) for the simultaneous detection of the following molecules: IL-2, 4, 5, 6, 9, 10, 13, 17A, 17F, 22, IFN-γ, and TNF-α, according to the manufacturer’s instructions.

### Flow cytometric analysis of cultured cells

Cells were washed and stained with the following fluorescent antibodies: PE anti-human PD-L1 (#12-5983-42, eBioscience), PE anti-mouse PD-L1 (#124308, BioLegend) and APC anti-human MHC class I (#311409, BioLegend). After incubation, the cells were washed with PBS containing 0.5% BSA and run on a BD FACSVerse flow cytometer (BD Biosciences). A LIVE/DEAD™ Fixable Far Red Dead Cell Stain Kit (#L34974, Invitrogen) was used to gate live cells. The results were analyzed using FlowJo software (Tree Star, Ashland, OR, USA). For flow cytometric analysis of PD-L1 induced by IFN-γ, cells were treated with 10 ng/ml human IFN-γ (#PHC4031, Gibco) for 24 h.

### Flow cytometric analysis of WNK3-knockdown human CD4^+^ and CD8^+^ T cells

For *WNK3* knockdown in human CD4^+^ and CD8^+^ T cells, live CD4^+^ and CD8^+^ T cells were sorted from human PBMCs using an MA900 sorter (SONY, San Jose, CA, USA) and stimulated with plate-bound anti-human CD3 (#BE0001-2, BioXCell, Lebanon, NH, USA)/anti-human CD28 (#BE0248, BioXCell) and 50 IU IL-2 for 24 h. These cells were spin-infected (900 × *g*, 90 min, 37 °C) with lentiviral supernatant (shNC and sh*WNK3*). Four days after lentiviral infection, cells were selected with 1 μg/ml puromycin for 7 days. Then, the cells were stimulated with Dynabeads Human T-Activator CD3/CD28 (Gibco) for 36 h. BD GolgiStop (#554724, BD Biosciences) was added for the last 6 h of stimulation. After stimulation, the cells were washed, and surface molecules were stained with anti-CD4 (OKT4, BioLegend) and anti-CD8 (SK1, BioLegend). Cells were then fixed with eBioscience/Invitrogen Intracellular (IC) Fixation Buffer (#00-8222-49, Invitrogen), washed with 1x Permeabilization Buffer (#00-8333-56, Invitrogen) and stained with antibodies for anti-perforin (B-D48, BioLegend) and anti-granzyme B (QA16A02, BioLegend).

### Survival analysis

Gene expression data for TCGA-lung cancer were downloaded using the TCGAbiolinks package (version 2.6.12), and preprocessed clinical data were downloaded from the TCGA pan-cancer clinical data resource^22^. The gene expression data and clinical data of the E-MTAB-923 cohort^23^ were downloaded from ArrayExpress (https://www.ebi.ac.uk/arrayexpress/experiments/E-MTAB-923/). Gene expression data and clinical information for colon (GSE39582) and gastric (GSE62254) cancers were obtained from GEO (https://www.ncbi.nlm.nih.gov/geo/).

Gene expression levels in multiple samples from the same patient were averaged to eliminate redundancy. Next, patients were separated into low- and high-expression groups based on the expression level of given genes. The optimal expression cutoffs were determined according to a previously described method^24^. A two-sided log-rank test was performed by using the survival package (version 2.42-6) in R.

### Immunoprecipitation and in vitro kinase assay

A total of 1.5 × 10^6^ HEK293FT cells seeded in 6-well plates were forward-transfected with 2.5 μg of plasmids encoding *WNK3* or *WNK3*-K159M using Lipofectamine 2000 (Invitrogen) and then harvested 72 h after transfection. Cells were lysed on ice in lysis buffer (50 mM Tris-HCl, pH 7.5, 1% NP-40, 130 mM NaCl, 10% glycerol, 10 mM MgCl_2_ and protease and phosphatase inhibitor cocktail (GenDEPOT, Katy, TX, USA)). Cell lysates (600 μg of protein) were incubated with 2 μl of anti-Myc antibody (#2276S, Cell Signaling Technology, Danvers, MA, USA) for 1 h with gentle rocking at 4 °C, followed by incubation with a 1:1 mixture of SureBeads Protein A and Protein G Magnetic Beads (#1614013, #1614023, Bio-Rad, Hercules, CA, USA). The precipitates were then washed three times in cold lysis buffer. For in vitro kinase assays, the beads containing anti-Myc-precipitated kinases were diluted in 15 μl of kinase dilution buffer X (#K20-09, SignalChem, British Columbia, Canada) and 50 μM DTT (#D86-09) and incubated with 10 μl of substrate/ATP mixture (1 μl of 10 mM ATP, 79 μl of kinase assay buffer III (#K03-09, SignalChem, mixed with DTT prior to use to a concentration of 250 μM), 80 μl of 0.5 mg/ml MBP (#M42-51N, SignalChem), and 1 μl of 1 M MnCl_2_ (#M40-09, SignalChem)) at ambient temperature for 50 min. ADP produced during a kinase reaction was measured using the ADP-Glo Kinase Assay (#V6930, Promega).

### Animal treatment and tumor challenges

All animal procedures for this study were approved by the Institutional Animal Care and Use Committee of Yonsei University College of Medicine (Seoul, Korea; 2019-0333). Before the experiments, the animals were acclimated for 7 days with 12-h light and dark cycles. C57BL/6 mice were purchased from SLC, Inc. (Shizuoka, Japan). Six- to eight-week-old male mice were subcutaneously injected with 5 × 10^5^ MC38 cells into the right flank on Day 0. For drug treatment, 5 mice per group were treated with a daily oral dose of 0, 5, or 10 mg/kg WNK463 (#CD00005886, Crysdot, Bel Air, MD, USA) from the point when the average tumor volume reached 50–200 mm^3^. For CD8^+^ T-cell depletion, mice were treated with 300 μg of anti-CD8 depleting antibody (clone 2.43, #BP0061, BioXCell) at Days -1, 3, 6, 10, and 13 via intraperitoneal injection. For a combination treatment of WNK463 and PD-1 blocking antibody, mice were treated with 10 μg of anti-PD-1 antibody (#BE0146, BioXCell) or IgG2a isotype control (#BE0089, BioXCell) at Day 8 via intraperitoneal injection and with a daily oral injection of 10 mg/kg WNK463 or vehicle from Day 8. Mice were randomly assigned to treatment groups and control (vehicle) groups and maintained under standardized conditions. Tumors were measured every day (length × width) from the first day of drug injection with a caliper. Tumor volume was calculated using the formula: 0.5 × major axis × minor axis^2^. Mice were treated with drugs daily for 9 days and sacrificed when the largest tumor volume reached 2 cm^3^. The individual mouse was considered to be an experimental unit. The sample size was sufficient to identify statistically significant differences between groups.

### Flow cytometric analysis of tumor cells

Excised tumors were digested in collagenase type IV and DNase at 37 °C for 20 min. Digested tissue was filtered through a 100-μm strainer to prepare a cell suspension. Then, the tumor cells and tumor-infiltrating lymphocytes (TILs) were purified by centrifugation (2000 rpm, 40 min, RT, brake off) through 40% and 80% discontinuous gradients of Percoll (GE Healthcare, Chicago, IL, USA).

Single-cell suspensions from TILs were prepared and stained with Fixable Viability Dye (Invitrogen) to label dead cells and with fluorochrome-conjugated antibodies. For surface staining, cells were washed with PBS and stained with the following antibodies diluted 1:400 in PBS: anti-CD4 (GK1.5, BioLegend), anti-CD8 (53-6.7, BioLegend), anti-CD45 (30-F11; BD Pharmingen, San Diego, CA, USA), anti-TCR (H57-597, BioLegend), anti-PD-L1 (10F.9G2, BioLegend), anti-PD1 (29F.1A12, BioLegend), and anti-TIM3 (RMT3-23, BioLegend). For intracellular transcription factor staining, cells were fixed with eBioscience/Invitrogen Foxp3 Fix/Perm Buffer, washed with eBioscience/Invitrogen Perm Buffer and stained with anti-TOX antibodies (REA473, Miltenyi Biotech). For intracellular cytokine staining, cells were stimulated with Cell Stimulation Cocktail plus protein transport inhibitors (00-4980-03, eBioscience/Invitrogen) for 6 h. After stimulation, the cells were washed, and surface molecules were stained. Cells were then fixed with eBioscience/Invitrogen Intracellular (IC) Fixation Buffer, washed with 1x Permeabilization Buffer (Invitrogen), and stained with the following antibodies diluted 1:200 in PBS: anti-TNF (MP6-XT22, BD Pharmingen), anti-IFN-γ (XMG1.2, BioLegend) and anti-granzyme B (QA16A02, BioLegend). A FITC Annexin V Apoptosis Detection Kit I (BD Pharmingen) was used to detect cellular apoptosis following the manufacturer’s instructions. Cell image acquisition was performed on a FACSCelesta (BD Biosciences). Data from the in vivo experiment were analyzed with GraphPad Prism version 9 (San Diego, CA, USA). A comparison between different groups was carried out using one-way analysis of variance (ANOVA), followed by a Tukey multiple comparison test.

## Results

### shRNA screening identifies druggable genes and cancer drivers that regulate PD-L1 expression

**Fig. 1:**
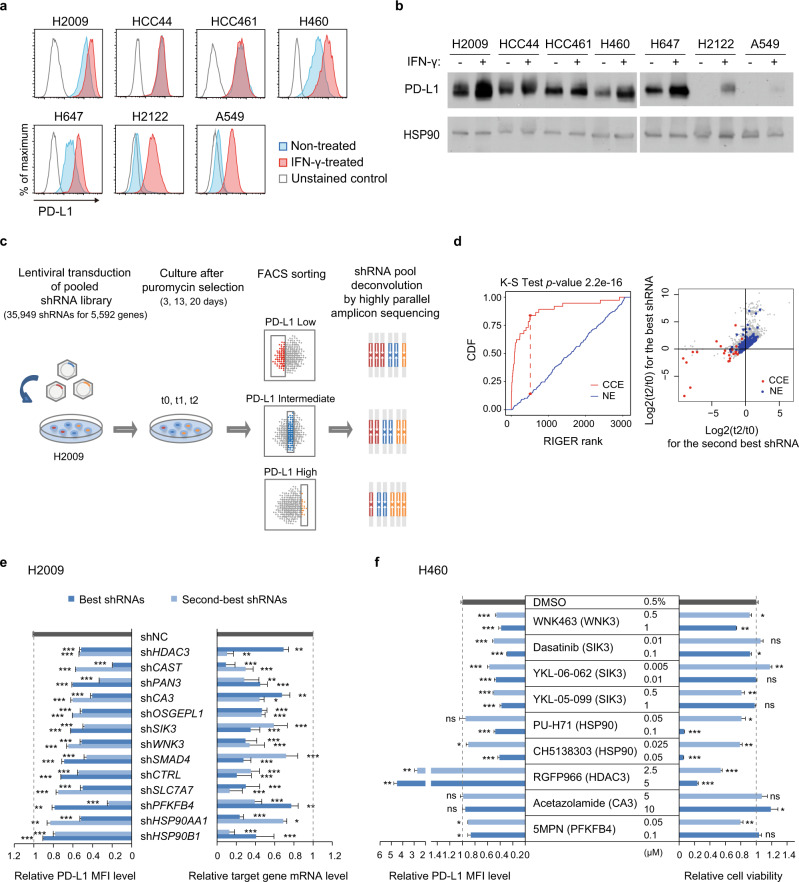
shRNA screening of druggable genes and cancer drivers that regulate PD-L1 expression. **a**, **b** PD-L1 membrane levels in NSCLC cell lines after 24 h of exposure to 10 ng/ml IFN-γ or nontreated control, analyzed by flow cytometry (**a**) and immunoblotting (**b**). HSP90 was used as a loading control. **c** Schematic workflow of the pooled shRNA screening. **d** Analysis of cells expressing shRNAs for core essential genes or nonessential genes at t2 compared to t0. (Left) A cumulative distribution function (CDF) plot of the RIGER rank distribution of “constitutive core essential” (CCE; red) or “nonessential” (NE; blue) gene groups (Hart T et al.)^25^. (Right) A scatter plot of genes showing the log_2_-fold changes (t2/t0) of the best (*y*-axis) and the second-best corresponding shRNAs (*x*-axis). **e** Genetic validation of positive PD-L1 regulators. The relative median fluorescence intensity (MFI) of PD-L1 in H2009 cells expressing shRNAs for hit genes vs. nontargeting negative control shRNA (shNC; black) is shown using bar plots on the left (*N* = 3). Target gene depletion by the corresponding shRNAs, measured by qRT‒PCR, is shown on the right (*N* = 3). The best (blue) and the second-best (light blue) shRNAs from the screening are shown for each hit gene. See Supplementary Fig. 1h for H460 data. **f** PD-L1 membrane expression levels and cell viability after drug treatment are shown using a bar plot. Relative quantification of the MFI of PD-L1 (left) and cell viability (right) of H460 cells compared to the DMSO vehicle control after five days of treatment with the indicated small molecule inhibitors (*N* = 3). Target genes are indicated in parentheses. See Supplementary Fig. 1i for H2009 data. Statistical differences were determined by a two-sided unpaired Student’s *t* test (**e**, **f**). *p < 0.05, **p < 0.01, ***p < 0.001, not significant (ns) *p* ≥ 0.05. Error bars indicate ±standard deviation.

We assembled a focused shRNA library (*N* = 35,949) to systematically identify druggable genes and cancer drivers that regulate PD-L1 expression. The druggable gene set included 5069 genes (32,643 shRNAs) associated with drugs or belonging to druggable targets such as kinases, receptors, and metabolic enzymes^9^ (Supplementary Table 1). The cancer driver gene set included the 800 most frequently mutated genes (5,611 shRNAs) in 13 major tumor types^10–17^ (Supplementary Table 2). Initial quality assessment revealed that the abundance of shRNAs in both libraries had close to normal distributions (Supplementary Fig. 1a). To select a suitable cell line for screening, we measured basal and IFN-γ-induced PD-L1 expression levels in seven non-small-cell lung cancer (NSCLC) cell lines (H2009, HCC44, HCC461, H460, H647, H2122, and A549) and chose H2009 due to it having the highest basal and IFN-γ-induced PD-L1 levels (Fig. 1a, b and Supplementary Fig. 1b, c). To focus on relatively less understood tumor-intrinsic PD-L1 regulatory mechanisms, we conducted screenings under IFN-γ untreated conditions. H2009 cells were transduced with lentiviral shRNAs (*N* = 35,949) and 32 nontargeting negative control shRNAs (Fig. 1c). After 3 days (t0), 13 days (t1), and 20 days (t2) of puromycin selection, FACS was performed to classify the cells into three distinct groups: PD-L1-low, PD-L1-intermediate, and PD-L1-high subpopulations (Fig. 1c and Supplementary Fig. 1d). Next, enriched and depleted shRNAs in each cell population were determined by highly parallel amplicon sequencing of the shRNA inserts (Fig. 1c). Finally, gene-level enrichment scores were calculated using the second-best method of the RNAi Gene Enrichment Ranking (RIGER) algorithm^21^ (Supplementary Fig. 1e). This method was adopted to observe phenotypic changes by at least two shRNAs because shRNAs have various on-target efficiencies and off-target effects. First, we evaluated the screening data quality by assessing the time-dependent depletion of the gold-standard constitutive core essential (CCE) genes^25^ using t0 and t2 cells without FACS sorting. CCE genes were significantly depleted at t2 compared to t0 (Kolmogorov‒Smirnov test, *p* = 2.2E-16), whereas the nonessential (NE) genes^25^ were not (Fig. 1d), supporting the high quality of our screening data. From the screening, 46 PD-L1 enhancers and 27 PD-L1 suppressors were identified from the PD-L1-low and PD-L1-high populations, respectively, consistently at t1 and t2 but not at t0 (Supplementary Fig. 1f and Supplementary Table 3). The identified targets included 40 druggable PD-L1 enhancers whose perturbation by associated drugs might downregulate PD-L1 expression and nine cancer drivers and seven cancer drivers that enhance or suppress PD-L1 expression, respectively (Supplementary Table 3).

Gene-set enrichment analysis of the 73 hit genes from the screening (46 PD-L1 enhancers and 27 PD-L1 suppressors) demonstrated that PD-L1 regulators are significantly involved in “Nucleotide excision repair” and “BMP/SMAD signaling” (*p* < 0.001; Supplementary Fig. 1g). The “Nucleotide excision repair” gene set included four PD-L1 suppressors (*POLR2C*, *PARP1*, *COPS5*, and *POLE4*; Supplementary Fig. 1g, left), which is consistent with the observations that defects in the DNA repair pathways upregulate PD-L1 expression via the neoantigen-T-cell activation-IFN-γ pathway and ATR-CHK1-dependent DNA damage signaling^26^. The “BMP/SMAD signaling” gene set included two PD-L1 enhancers (*BMPR2* and *SMAD4*) and one PD-L1 suppressor (*UBE2D3*; Supplementary Fig. 1g, right). As ubiquitin conjugating enzyme E2 D3 (*UBE2D3*) inhibits SMAD signaling triggered by BMPs-BMPRs^27,28^, this finding suggests that BMP signaling may participate in PD-L1 upregulation.

Our study identified PD-L1 enhancers, which could be direct therapeutic targets, with more robust statistical evidence than that for PD-L1 suppressors. Hence, further analyses were conducted focusing on the PD-L1 enhancers.

### Genetic and chemical validation reveals WNK3 and SIK3 to be positive PD-L1 regulators

Next, we validated the PD-L1 modulating function of the 46 PD-L1 enhancers with cell lines (H2009 and H460) generated to stably express the most efficient and the second-most efficient PD-L1 depleting shRNAs per candidate gene. Of these, 13 gene knockdowns (*HDAC3*, *CAST*, *PAN3*, *CA3*, *OSGEPL1, SIK3*, *WNK3*, *SMAD4*, *CTRL*, *SLC7A7*, *PFKFB4*, *HSP90AA1*, and *HSP90B1*) most robustly reduced membrane PD-L1 levels, as demonstrated by flow cytometry and qPCR analysis (Fig. 1e and Supplementary Fig. 1h). Among these 13 PD-L1 enhancers, except for the two cancer drivers (*PAN3* and *SMAD4*), 11 of the enhancers belong to druggable genes. In particular, seven of the druggable genes (*WNK3*, *SIK3*, *HSP90AA1*, *HSP90B1*, *HDAC3*, *CA3*, and *PFKB4*) were eligible for chemical validation due to the presence of commercially available small molecule inhibitors (Supplementary Table 4). Of these, the WNK3, SIK3, HSP90AA1, and HSP90B1 inhibitors decreased membrane PD-L1 levels in both H460 and H2009 cells, and only the WNK3 (WNK463) and SIK3 inhibitors (dasatinib, YKL-06-062, and YKL-05-099) reduced membrane PD-L1 levels at concentrations that did not profoundly affect cell viability (>60%; Fig. 1f and Supplementary Fig. 1i). Unexpectedly, the HDAC3 inhibitor RGFP966 increased PD-L1 levels in both H460 and H2009 cells (Fig. 1f and Supplementary Fig. 1i), which was inconsistent with the genetic depletion, possibly due to an off-target effect of either sh*HDAC3* or RGFP966.

Our data collectively indicated that WNK3 and SIK3 were positive PD-L1 regulators in lung cancer cells. Furthermore, inhibition of these genes suppressed membrane PD-L1 levels without causing severe cytotoxicity to H460 and H2009 cells. Thus, these two genes were subjected to further functional analyses.

### Genetic and chemical validation reveals the immune-modulatory effect of WNK3 under coculture conditions

Before evaluating WNK3 and SIK3, we first confirmed that H460 and H2009 cells stably expressing sh*PD-L1* (Supplementary Fig. 2a, c) exhibited increased susceptibility (decreased cancer cell viability) to IL-2 and anti-CD3-activated peripheral blood mononuclear cells (PBMCs; Supplementary Fig. 2b, d).

**Fig. 2:**
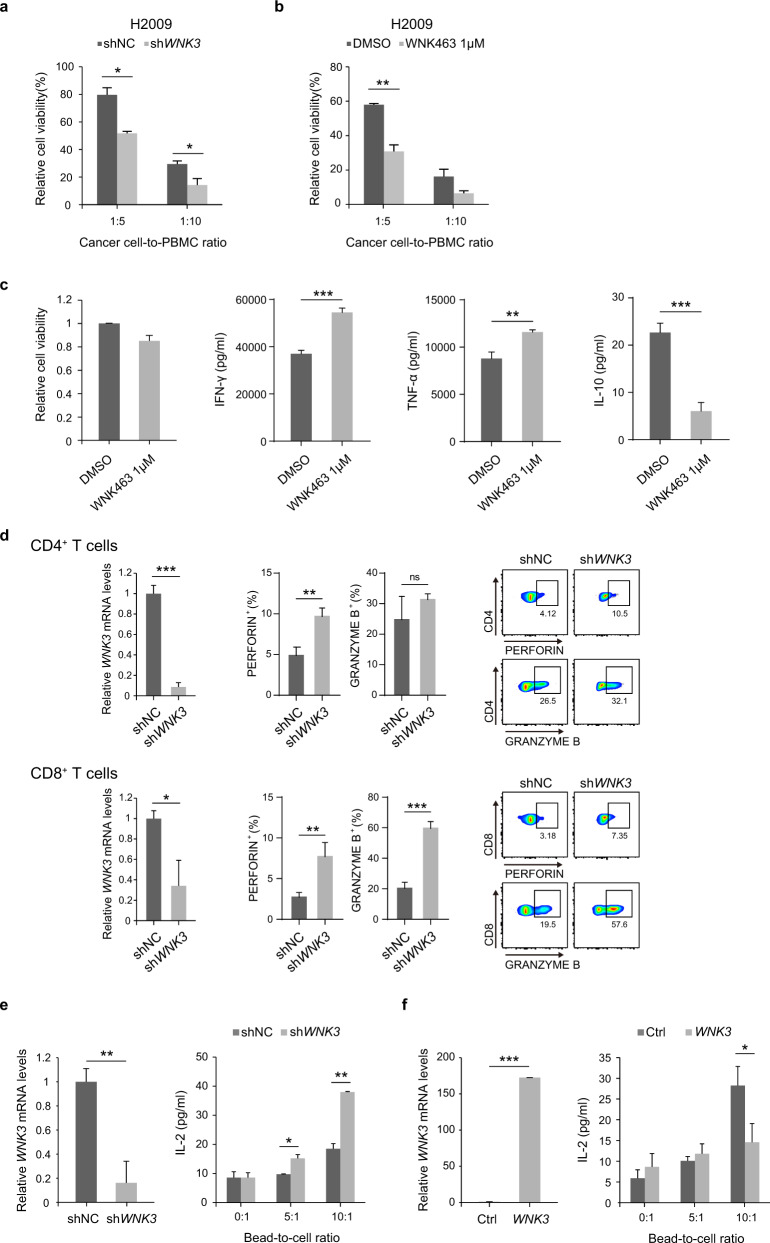
Genetic and chemical validation of the immune-modulatory effect of the candidate genes in in vitro coculture conditions. **a** Relative viability of H2009 cells expressing sh*WNK3* or shNC after 72 h of coculture with activated PBMCs (*N* = 3). **b** Relative viability of H2009 cells cocultured with activated PBMCs with 1 µM WNK463 or DMSO for 48 h. H2009 cells were pretreated with 1 µM WNK463 or DMSO vehicle for nine days (*N* = 3). **c** Cell viability of PBMCs after 72 h of treatment with WNK463 or DMSO vehicle control (*N* = 2) (left). Abundance of released cytokines from PBMCs after 72 h of treatment with 1 µM WNK463 or DMSO vehicle control (*N* = 3) (right). **d** Perforin and granzyme B production in human CD4^+^ T cells (top) or CD8^+^ T cells (bottom) expressing sh*WNK3* or shNC after 36 h of TCR stimulation (*N* = 3). *WNK3* depletion was measured by qRT‒PCR (*N* = 3). **e** IL-2 concentrations measured by ELISA in supernatants obtained from Jurkat T cells expressing sh*WNK3* or shNC after 48 h of stimulation with anti-CD3/CD28 beads at different bead-to-cell ratios (5:1, 10:1) (*N* = 3, right). *WNK3* depletion in Jurkat T cells was measured by qRT‒PCR (*N* = 3, left). **f** IL-2 concentrations measured by ELISA in supernatants obtained from Jurkat T cells transduced with lentiviral vectors expressing the *WNK3* transgene or with empty vectors after 48 h of stimulation with anti-CD3/CD28 beads at different bead-to-cell ratios (5:1, 10:1) (*N* = 3, right). *WNK3* overexpression in Jurkat T cells was measured by qRT‒PCR (*N* = 3, left). Statistical differences were determined by a two-sided unpaired Student’s *t* test. *p < 0.05, **p < 0.01, ***p < 0.001, not significant (ns) *p* ≥ 0.05. Error bars indicate ±standard deviation (**a**, **b**, **c**, **e**, **f**) or ±standard error of the mean (**d**).

Next, we examined whether shRNA-mediated depletion of *WNK3* could sensitize cancer cells to immune cell attack. We observed increased death of sh*WNK3* cells in the presence of activated PBMCs compared to those without PBMCs (Fig. 2a). Likewise, treatment with WNK463, a pan-WNK inhibitor, in the coculture condition significantly increased the death of cancer cells by activated PBMCs (Fig. 2b). This WNK463-mediated anticancer effect of activated PBMCs was partially reversed by *PD-L1* overexpression in cancer cells (Supplementary Fig. 2e, f), suggesting that PD-L1 depletion in cancer cells at least partially contributes to enhanced anticancer immunity. Intriguingly, we found that treatment of PBMCs with WNK463 significantly enhanced the production of immune-activating cytokines, including IFN-γ and TNF-α, but suppressed immunosuppressive cytokines, such as IL-10, at a concentration that did not affect the viability of PBMCs (Fig. 2c), suggesting the dual antitumor mechanisms of WNK463 for PD-L1 suppression on cancer cells together with immune cell activation. To further characterize how WNK463 promotes immune cell activation, we isolated CD4^+^ or CD8^+^ T cells from mouse spleens and stimulated them in the presence or absence of WNK463. WNK463 was sufficient to boost perforin and granzyme B production in both CD4^+^ and CD8^+^ T cells in comparison to that with vehicle treatment (Supplementary Fig. 2g). This result implied that WNKs play intrinsic functions in T-cell activation. We further demonstrated that the production of perforin and granzyme B was considerably boosted by an effective knockout of *Wnk3* in murine CD4^+^ and CD8^+^ T cells (Supplementary Fig. 2h). Human CD4^+^ T cells and CD8^+^ T cells consistently generated considerably more perforin and/or granzyme B when *WNK3* was knocked down by shRNA compared to control shRNA (Fig. 2d). Furthermore, Jurkat T cells stably expressing sh*WNK3* or overexpressing *WNK3* also displayed significantly enhanced or suppressed IL-2 secretion, respectively (Fig. 2e, f). These findings collectively demonstrated that WNK3 has intrinsic T-cell functions that inhibit T-cell activation. Next, we examined how WNK3 inhibits T-cell activation. Given that WNK3 possesses kinase activity and that PI3K-AKT signaling is crucial for the production of granzyme B^29^, we hypothesized that WNK3 would primarily influence proximal TCR signaling cascades by modulating AKT pathways. Indeed, compared to control sgRNA and vehicle, CRISPR/Cas9-based knockout of *Wnk3* and WNK463 treatment were sufficient to increase the phosphorylation of AKT and pS6, particularly in CD8^+^ T cells and potentially in CD4^+^ T cells (Supplementary Fig. 2i, j).

In contrast, SIK3 inhibition had the opposite effect on immune cells. Although sh*SIK3* and SIK3 inhibitors decreased PD-L1 levels in cancer cells (Fig. 1e, f and Supplementary Fig. 1h, i), a SIK3 inhibitor, YKL-06-062, rendered the cancer cells resistant to the immune cells in the coculture condition (Supplementary Fig. 2k). Consistent with this observation, multiple SIK3 inhibitors (dasatinib and YKL-06-062) significantly decreased granzyme B release by PBMCs at concentrations that did not affect the viability of PBMCs (Supplementary Fig. 2l), suggesting that SIK3 inhibition has a cancer cell-independent anti-immunogenic effect.

These data collectively indicated a dual role of WNK3 inhibition in the coculture condition. WNK3 inhibition suppressed PD-L1 expression in cancer cells, thereby increasing their susceptibility to CD8^+^ T cells, whereas in immune cells, it facilitated antitumorigenic cytokine secretion by CD8^+^ T cells.

### WNK3 is a transcriptional regulator of *PD-L1*

**Fig. 3:**
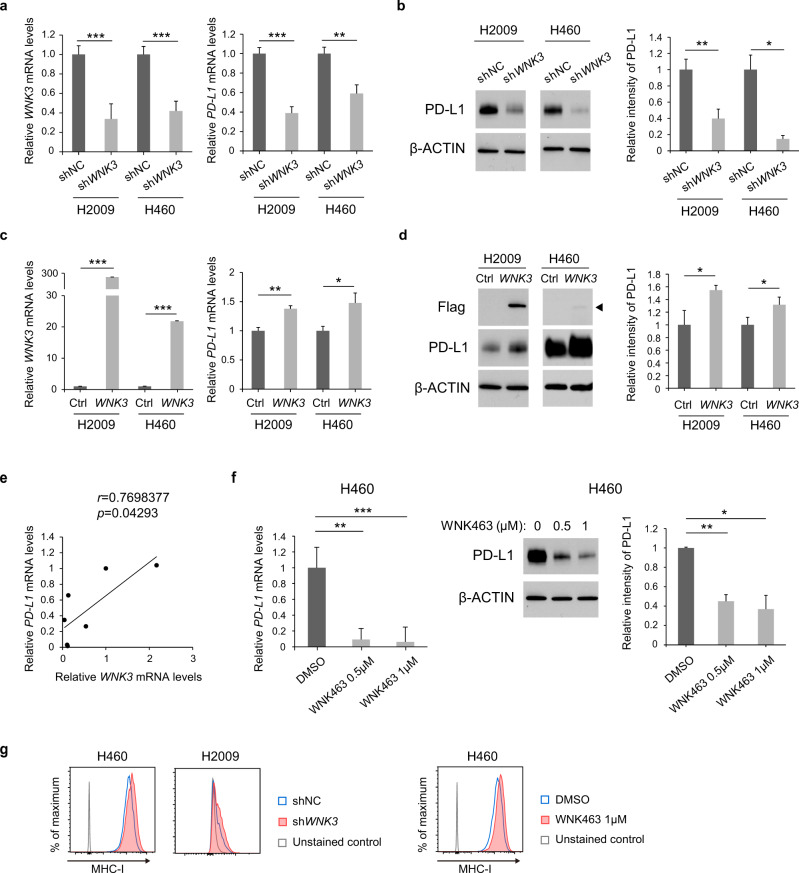
WNK3 is a transcriptional regulator of *PD-L1*. **a** *PD-L1* mRNA levels in *WNK3* knockdown cells. The mRNA levels of *WNK3* (left) and *PD-L1* (right) in H2009 and H460 cells transduced with the indicated shRNAs were determined by qRT‒PCR (*N* = 3). **b** (Left) Effect of *WNK3* knockdown on the PD-L1 protein levels assessed by immunoblot of whole-cell lysates from H2009 and H460 cell lines. (Right) Bar plots represent the quantitative densitometric analysis of PD-L1 immunoblots (*N* = 3). β-Actin was used as a loading control. **c** *PD-L1* mRNA levels in *WNK3*-overexpressing cells. mRNA levels of *WNK3* (left) and *PD-L1* (right) in H2009 cells and H460 cells transfected with *WNK3* cDNA plasmids or with empty vectors were determined by qRT‒PCR (*N* = 3). **d** (Left) Effect of *WNK3* overexpression on the expression of the indicated proteins assessed by immunoblot of whole-cell lysates from H2009 and H460 cell lines. (Right) Bar plots represent the quantitative densitometric analysis of PD-L1 immunoblots (*N* = 3). β-Actin was used as a loading control. **e** Correlation between *WNK3* and *PD-L1* expression in the seven NSCLC cell lines in Fig. 1a, b. determined by Pearson’s correlation coefficient. **f** *PD-L1* mRNA (left) and protein (right) levels in H460 cells after WNK463 treatment with the indicated concentrations were determined by qRT‒PCR and immunoblotting, respectively (*N* = 3). Bar plots (right) represent the quantitative densitometric analysis of PD-L1 immunoblots (*N* = 3). β-Actin was used as a loading control. **g** Flow cytometry analysis of MHC class I on H460 and H2009 cells expressing sh*WNK3* or shNC (left) and on H460 cells after 72 h of WNK463 treatment (right). Statistical differences were determined by a two-sided unpaired Student’s *t* test. *p < 0.05, **p < 0.01, ***p < 0.001. Error bars indicate ±standard deviation.

Next, we examined whether PD-L1 regulation by WNK3 is achieved at the mRNA or protein level with H2009 and H460 cell lines that had been generated to express sh*WNK3* or to overexpress *WNK3*. Both the mRNA and protein levels of PD-L1 were decreased in the two sh*WNK3*-expressing cell lines (Fig. 3a, b), and again, increases in both the mRNA and protein levels of PD-L1 were observed in the two *WNK3*-overexpressing cell lines (Fig. 3c, d). We deduced that sh*WNK3*-mediated *PD-L1* downregulation was an on-target effect since ectopic expression of *WNK3* was sufficient to rescue the reduced PD-L1 levels in cells expressing sh*WNK3* targeting its 3′ UTR (Supplementary Fig. 3a). Moreover, the expression of *WNK3* and *PD-L1* showed a significant positive correlation in the panel of seven NSCLC cell lines (Fig. 3e). Together, these results indicated that WNK3 transcriptionally regulates PD-L1 expression. Consistent with the genetic data, the pan-WNK inhibitor WNK463 depleted PD-L1 mRNA and protein levels (Figs. 1f and 3f and Supplementary Fig. 1i). As WNK463 is a pan-WNK inhibitor, we explored the ability of other WNK family members (*WNK1*, *WNK2*, and *WNK4*) to regulate PD-L1 expression. Unlike *WNK3*, shRNA-mediated knockdown of *WNK1* and *WNK2* and siRNA-mediated knockdown of *WNK4* did not affect PD-L1 expression (Supplementary Fig. 3b). Moreover, overexpression of *WNK1*, *WNK2*, or *WNK4* did not affect PD-L1 levels in H2009 cells (Supplementary Fig. 3c). The lack of effect of other WNK family members on PD-L1 expression indicated that WNK463 might regulate PD-L1 through WNK3 inhibition. Notably, WNK3 inhibition did not affect MHC class I protein expression, indicating that it did not impair antigen presentation by cancer cells to CD8^+^ T cells (Fig. 3g). These data collectively demonstrated that WNK3 is a necessary and sufficient factor regulating *PD-L1* transcription.

### WNK3 promotes PD-L1 expression through the JNK/c-JUN pathway

**Fig. 4:**
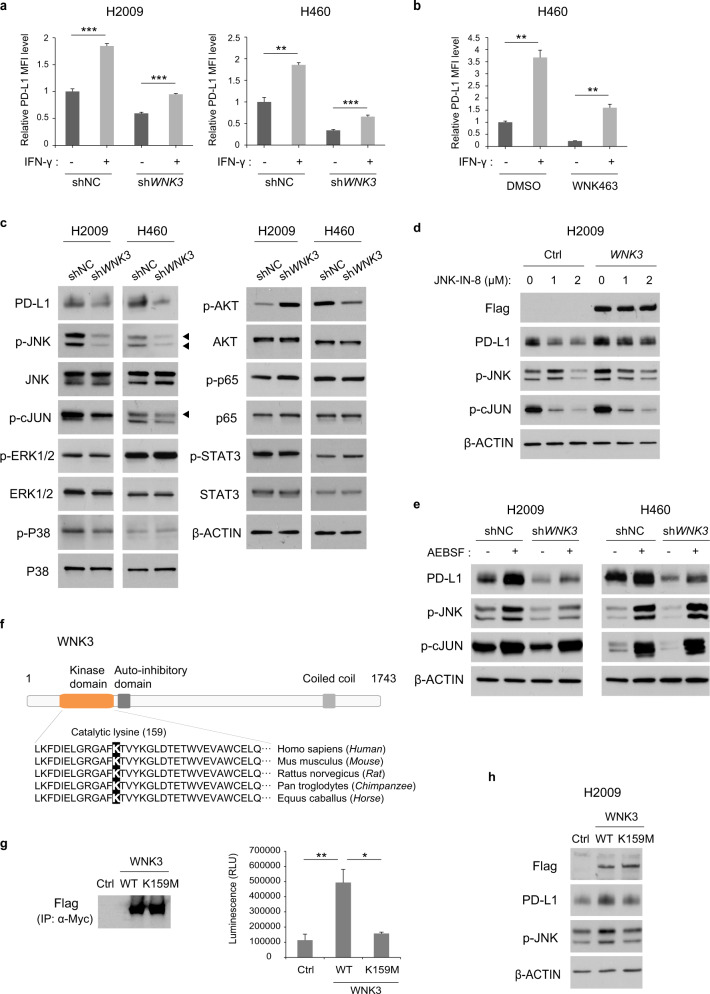
WNK3 sustains PD-L1 expression through the JNK/c-JUN pathway. **a**, **b** PD-L1 membrane levels in the indicated cell lines after treatment with IFN-γ (10 ng/ml) or medium alone were analyzed by flow cytometry (*N* = 3). Cells were transduced with the indicated shRNAs (**a**) or were treated with 1 μM WNK463 (**b**). **c** Effect of *WNK3* knockdown on the phosphorylation levels of known PD-L1 transcription regulators (JNK, c-JUN, ERK, P38, AKT, p65, and STAT3) assessed by immunoblotting of whole-cell lysates from the H2009 and H460 cell lines. Differentially expressed proteins are marked with arrowheads. β-Actin was used as a loading control. **d** Consequence of JNK inhibition by JNK-IN-8 on PD-L1 levels in H2009 cells transfected with *WNK3* cDNA plasmids or with empty vectors assessed by immunoblot. H2009 cells were treated with the indicated concentrations of JNK-IN-8 for 48 h. β-Actin was used as a loading control. **e** Effect of JNK activation by AEBSF on PD-L1 levels in H2009 and H460 cells expressing sh*WNK3* or nontargeting shRNA assessed by immunoblot. H2009 cells were treated with 100 μM AEBSF for 24 h, and H460 cells were treated with 250 μM AEBSF for 8 h. β-Actin was used as a loading control. **f** Schematic representation of the WNK3 protein domains. The highly conserved catalytic lysine (Lys 159) in the kinase domain is highlighted in black. **g** In vitro protein kinase activity of wild-type WNK3 and WNK3 K159M. HEK293 cells were transfected with *WNK3*-Myc-Flag (WT), *WNK3*-K159M-Myc-Flag (K159M), or empty pCMV6-Myc-Flag vector (Ctrl). The immunoprecipitates obtained with anti-Myc antibody were incubated with the substrate, ATP and cofactors and assessed by the ADP-Glo kinase assay (right). The protein levels of immunoprecipitated WNK3 were assessed by immunoblotting (left) (*N* = 3). **h** Effect of transfection of the plasmids encoding *WNK3* wild type, *WNK3* K159M, and the negative control in H2009 cells on the expression of the indicated proteins assessed by immunoblot. β-actin was used as a loading control. Statistical differences were determined by a two-sided unpaired Student’s *t* test (**a**, **b**, **g**). *p < 0.05, **p < 0.01, ***p < 0.001. Error bars indicate ±standard deviation.

To determine whether WNK3-dependent PD-L1 transactivation is coupled to the IFN-γ pathway, we measured membrane PD-L1 levels in *WNK3*-depleted or WNK3-inhibited cells in the presence or absence of supplemented IFN-γ. We hypothesized that supplementing IFN-γ might not rescue the decreased PD-L1 expression by *WNK3* depletion or inhibition if WNK3 functions downstream of the IFN-γ signaling pathway. However, the reduction in PD-L1 levels was rescued by recombinant human IFN-γ, suggesting that WNK3 functions independently of the IFN-γ signaling pathway (Fig. 4a, b). To elucidate the signaling pathways involved in WNK3-mediated PD-L1 regulation, we investigated several key signaling pathways, including JNK/c-JUN, ERK, p38, AKT, NF-κB, and JAK/STAT, which regulate PD-L1 transcription in cancer cells^30^. We found that only phospho-JNK and phospho-c-JUN were downregulated by *WNK3* knockdown (Fig. 4c), suggesting that WNK3 is an upstream regulator of the JNK signaling pathway. Moreover, enhanced PD-L1 expression by *WNK3* overexpression was reduced with the JNK inhibitor JNK-IN-8 (Fig. 4d), whereas JNK activation by 4-(2-aminoethyl)benzenesulfonyl fluoride hydrochloride (AEBSF) at least partially rescued PD-L1 expression in *WNK3*-knockdown cells (Fig. 4e). Next, to determine whether the WNK3-mediated modulation of PD-L1 levels depends on the kinase activity of WNK3, we evaluated the PD-L1 regulatory capacity of a WNK3 kinase-dead mutant^31,32^ (WNK3-K159M) (Fig. 4f). The immunoprecipitated kinase-dead WNK3-K159M mutant showed no significant kinase activity, whereas WT WNK3 phosphorylated the substrate myelin basic protein (MBP) (Fig. 4g). The WNK3-K159M mutant failed to elevate PD-L1 expression levels as much as WT WNK3 (Fig. 4h), showing that PD-L1 regulation is at least partially mediated by WNK3 kinase activity. Phosphorylated JNK levels were also correlated with WNK3 kinase activity (Fig. 4h). These data demonstrated that WNK3 promoted PD-L1 expression through the JNK/c-JUN pathway.

### WNK463 suppresses neoplastic PD-L1 and activates CD8^+^ T cells to regress MC38 tumor growth

**Fig. 5:**
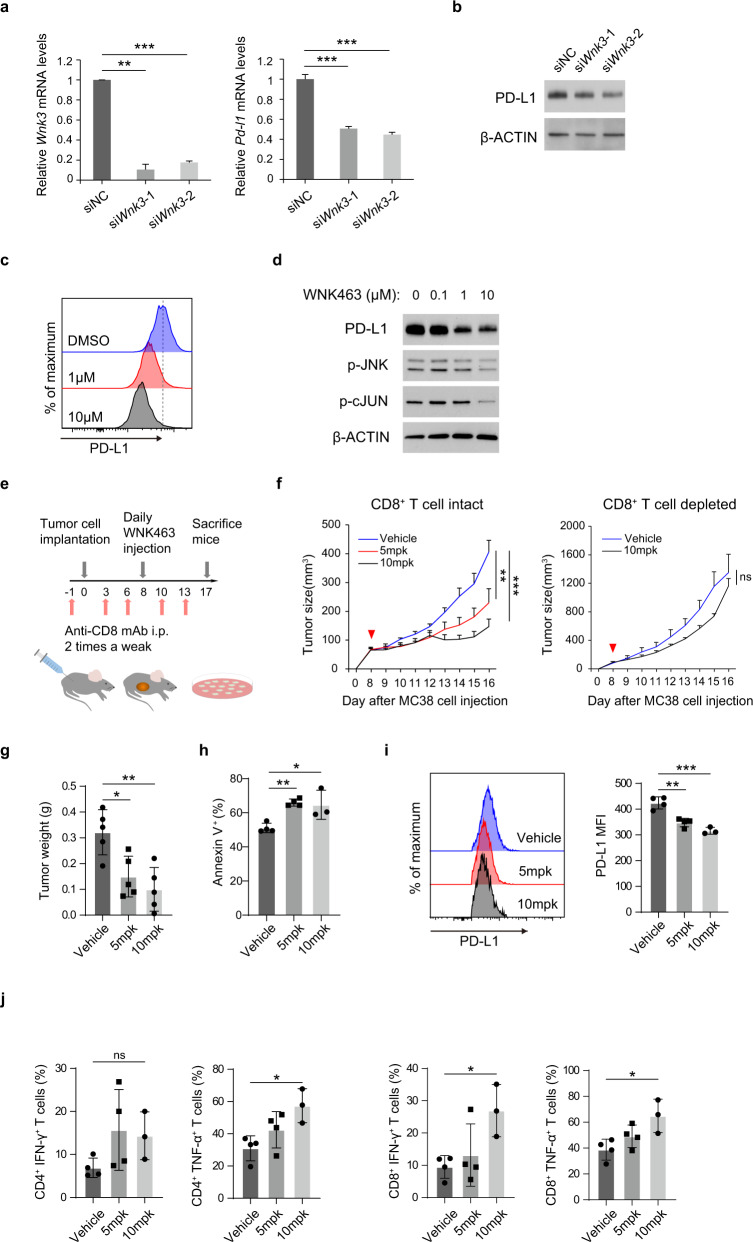
Genetic and chemical inhibition of WNK3 sensitizes PD-L1-dependent syngeneic tumors to anti-PD-1 therapy. **a** *Pd-l1* expression levels in *Wnk3*-depleted MC38 cells. The mRNA levels of *Wnk*3 (left) and *Pd-l1* (right) in MC38 cells transfected with si*Wnk*3 or control siRNA were determined by qRT‒PCR (*N* = 3). **b** Effect of *Wnk3* knockdown on the PD-L1 protein levels assessed by immunoblot of whole-cell lysates from MC38 cells. β-Actin was used as a loading control. **c** PD-L1 membrane levels on MC38 cells after 72 h of WNK463 treatment with the indicated concentrations were examined by flow cytometry. **d** Effect of WNK463 treatment at different concentrations in MC38 cells assessed by immunoblot with the indicated antibodies. β-Actin was used as a loading control. **e** Schematic diagram of the in vivo experimental protocol. **f** Effect of WNK463 treatment on MC38 tumor growth with (right) or without (left) combined treatment with CD8 antibody. Tumor volume was measured at the indicated time points. Arrowheads indicate the first day of WNK463 injection. *n* = 5 mice per group. **g** Weights of MC38 tumors obtained from CD8^+^ T-cell competent mice (**f**, left) at the endpoint were measured. **h** Apoptosis of extracted MC38 tumor cells (**f**, left) was assessed by Annexin-V staining. **i** PD-L1 levels in extracted MC38 tumor cells (**f**, left) were evaluated by flow cytometry. **j** IFN-γ and TNF-α produced by tumor-infiltrating CD4^+^ and CD8^+^ T cells (**f**, left) were analyzed by flow cytometry. Cells were pregated on live CD45^+^TCRb^+^ cells. Statistical significance was determined by a two-sided unpaired Student’s *t* test (**a**), two-way ANOVA (**f**) or an ordinary one-way ANOVA followed by the Tukey multiple comparison test with a single pooled variance (**g**–**j**). *p < 0.05, **p < 0.01, ***p < 0.001, not significant (ns) *p* ≥ 0.05. Error bars indicate ±standard deviation (**a**, **g**–**j**) or ±standard error of the mean (**f**).

The human colon cancer cell line HCT116 also recapitulated the WNK3/PD-L1 relationship (Supplementary Fig. 4a, b), suggesting that this axis might be functional in cancers other than NSCLC. To evaluate the functional role of the WNK3/PD-L1 axis in an in vivo setting, we used the MC38 syngeneic colon adenocarcinoma mouse model, one of the commonly used mouse xenograft models responsive to anti-PD-1 or anti-PD-L1 monotherapy^33^. Importantly, similar to human NSCLC cell lines, MC38 had an intact WNK3/PD-L1 axis, as evidenced by the si*Wnk3*-mediated depletion of Pd-l1 at the mRNA and protein levels (Fig. 5a, b). Furthermore, the pan-WNK inhibitor WNK463 reduced PD-L1 expression in MC38 cells (Fig. 5c, d). Oral gavage of WNK463 (0, 5, or 10 mgkg^−1^; once a day) in C57BL/6 mice carrying MC38 tumors (Fig. 5e) showed a dose-dependent suppression of tumor growth (Fig. 5f, left, and 5g). Consistently, WNK463 treatment promoted apoptosis in neoplastic cells (Fig. 5h). However, no significant tumor regression by WNK463 treatment was found in CD8^+^ T-cell-depleted mice (Supplementary Fig. 4c and Fig. 5f, right), although PD-L1 expression consistently decreased in this context (Supplementary Fig. 4d), indicating the crucial roles of CD8^+^ T cells in WNK463-mediated antitumor activity. Importantly, flow cytometry analysis of the tumors obtained after WNK463 therapy (Fig. 5f, left) revealed a dose-dependent decrease in PD-L1 levels in neoplastic cell populations (Fig. 5i) but an increase in IFN-γ and TNF-α produced by CD4^+^ and CD8^+^ T cells in tumor-infiltrating lymphocytes (Fig. 5j). These data collectively demonstrated that WNK3 inhibition promoted CD8^+^ T-cell-mediated antitumor immunity through neoplastic PD-L1 suppression and immune cell activation.

### The synergistic effect of WNK463 and PD-1 blockade combination treatment is dependent on the cytotoxicity of CD8^+^ T cells in vivo

**Fig. 6:**
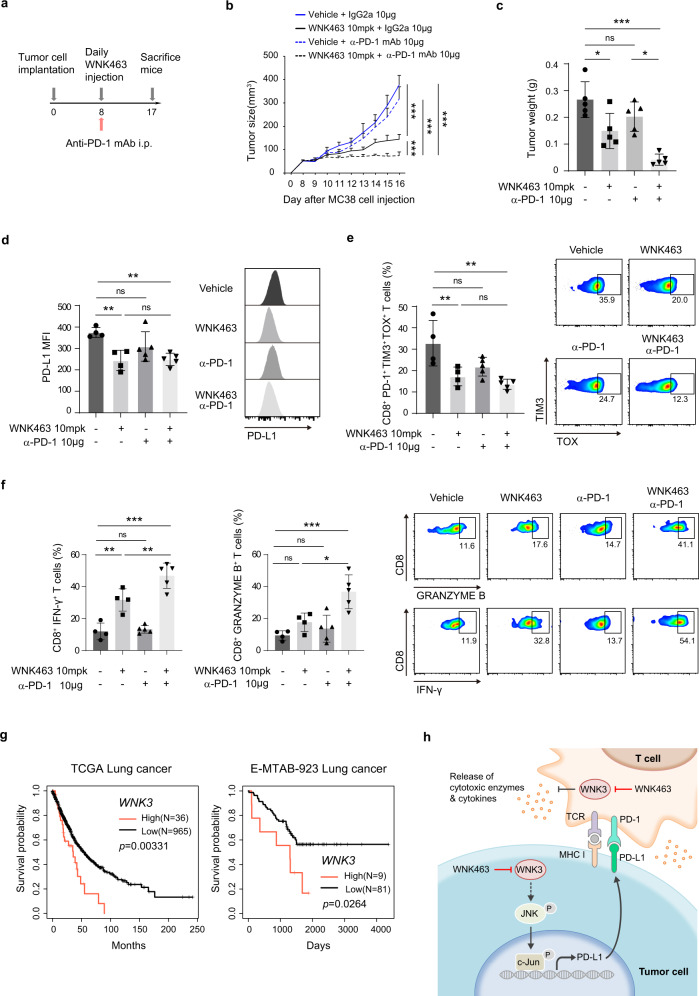
The combination of WNK463 and PD-1 blockade synergistically suppresses tumor growth in vivo. **a** Schematic diagram of the in vivo experimental protocol. **b** Effect of combination treatment with WNK463 and anti-PD-1 antibody on MC38 tumor growth. Tumor volume was measured at the indicated time points. *n* = 5 mice per group. **c** Weights of MC38 tumors obtained from mice (**b**) at the endpoint were measured. **d** PD-L1 levels in extracted MC38 tumor cells (**b**) were evaluated by flow cytometry. **e** Fractions of CD8^+^, PD-1^+^, TIM3^+^, and TOX^+^ T cells out of the total CD8^+^ T cells in the tumors obtained from **b** were evaluated by flow cytometry. **f** IFN-γ and granzyme B produced by tumor-infiltrating CD8^+^ T cells (**b**) were analyzed by flow cytometry. **g** Kaplan‒Meier survival analysis of overall survival for lung cancer patients with high vs. low *WNK3* expression in the TCGA (left) and E-MTAB-923 (right) cohorts. Patients were classified based on the optimal gene expression thresholds^24^. **h** A schematic summary of the antitumor mechanism of WNK3 inhibition. Statistical significance was determined by two-way ANOVA (**b**), ordinary one-way ANOVA followed by the Tukey multiple comparison test with a single pooled variance (**c**–**f**), or the log-rank test (**g**). *p < 0.05, **p < 0.01, ***p < 0.001, not significant (ns) *p* ≥ 0.05. Error bars indicate ±standard deviation (**c**–**f**) or ±standard error of the mean (**b**).

Since in vivo WNK463 treatment suppresses tumor growth by reducing PD-L1 expression, we hypothesized that WNK463 may enhance the therapeutic efficacy of a low-dose PD-1 blockade. Mice bearing subcutaneous MC38 tumors were treated with WNK463 (10 mg/kg), anti-PD-1 antibody (10 μg) or both (Fig. 6a). In contrast to monotherapy with a low-dose anti-PD-1 antibody (10 μg) that did not directly affect tumor growth, combined treatment with WNK463 completely blocked tumor growth (Fig. 6b) and showed a marked decrease in tumor weights (Fig. 6c). As expected, PD-L1 levels in neoplastic cell populations were reduced in the WNK463-treated groups (Fig. 6d). Consistent with this observation, the cotreatment group demonstrated the most significant reduction in CD8^+^, PD-1^+^, TIM3^+^, and TOX^+^ exhausted T cells (Fig. 6e), indicating enhanced activity of tumor-infiltrated CD8^+^ T cells. Additionally, cytotoxic CD8^**+**^ T cells producing IFN-γ and granzyme B were markedly increased in tumors cotreated with WNK463 and anti-PD-1 (Fig. 6f). Overall, these results demonstrate that WNK463 in combination with low-dose anti-PD-1 therapy has a synergistic effect on the activation of tumor-infiltrating CD8^+^ T cells and on suppressing tumor growth.

To finally assess the clinical implications of WNK3 in tumor immune resistance, we compared the prognosis of lung cancer patients with *WNK3*-high and *WNK3*-low expression tumors. In both the TCGA and E-MTAB-923 cohorts, 3.6% to 10% of patients with the highest *WNK3* expression exhibited a poorer prognosis and thus shorter overall survival than those with lower *WNK3* expression (Fig. 6g). Consistently, *WNK3* expression was significantly associated with poor overall survival in colon (GSE39582) and gastric (GSE62254) cancer cohorts (Supplementary Fig. 5a). This observation suggested that the worse prognosis of the *WNK3*-high tumors might be due to suppressed antitumor immunity caused by WNK3.

## Discussion

PD-1/PD-L1 is an immune checkpoint leading to T-cell apoptosis, anergy, and functional exhaustion^2^. Blocking the PD-1/PD-L1 axis expands and restores the effector function of CD8^+^ T cells for effective antitumor immune responses and tumor rejection^2^. Therefore, the identification of novel PD-L1 regulators, understanding their molecular regulatory mechanisms and developing their inhibition strategies offer unique therapeutic opportunities. In this study, we performed pooled shRNA screening to systematically identify PD-L1 enhancers by focusing on 5,069 druggable genes and 800 cancer drivers. Eleven druggable genes (*WNK3*, *HSP90AA1*, *CA3*, *CAST*, *PFKFB4*, *SLC7A7*, *HDAC3*, *HSP90B1*, *SIK3*, *CTRL*, and *OSGEPL1*) and two cancer drivers (*SMAD4* and *PAN3*) were finally identified and validated to enhance PD-L1 expression. Of these, HSP90, HDAC3, and SMAD4 were previously recognized as PD-L1 regulators. Specifically, HSP90 inhibition decreases PD-L1 and PD-L2 expression in tumor cells^34^ and makes immune-refractory tumors susceptible to adoptive T-cell transfer and PD-1 blockade immunotherapies^35^. HDAC3 has bidirectional effects on PD-L1 expression and acts by either decreasing histone H3 acetylation in the PD-L1 promoter^36^ or activating STAT3 signaling^37^. SMAD4 was previously identified as a positive PD-L1 regulator by CRISPR‒Cas9 screening^7^. In addition, activation of TGF-β signaling leads to complex formation of SMAD4 with SMAD2 and SMAD3, which subsequently enhances PD-L1 expression^38^. The rest of the hit genes are not yet known to be PD-L1 enhancers to our knowledge. PD-L1-enhancing oncogenes, such as *EGFR*, *ALK*, *MET*, *AKT*, *MYC*, and *CDK5*, were not identified as hits in this study because *KRAS* mutations in lung cancer cell lines (H2009 and H460) could establish distinct PD-L1 regulatory axes. Among the novel PD-L1 enhancers, we demonstrated that WNK3, a less characterized protein in cancer, is necessary and sufficient for the transactivation of PD-L1 through its kinase activity in JNK-dependent but IFN-γ/STAT3-independent mechanisms. The WNK3/PD-L1 axis is preserved across various cancer types and species. Ablation of *WNK3* and small-molecule-mediated chemical inhibition of pan-WNK kinase activity elicited PD-L1 downregulation and antitumor activity in a CD8^+^ T-cell-dependent manner (Fig. 6h). These findings highlight WNK3 as a novel therapeutic target to enhance antitumor immunity.

WNK protein kinases (WNK1, WNK2, WNK3, and WNK4) belong to the ‘with no lysine’ family of serine-threonine kinases and are characterized by the lack of catalytic lysine in subdomain II, which is required for ATP binding in most protein kinases. Instead, WNK family proteins have a conserved catalytic lysine in subdomain I^39^. Aldosterone^40^, angiotensin II^41^, vasopressin^42^, insulin^43^, dietary salt, and potassium intake^44^ are upstream regulators of the WNK signaling cascade in the kidney. WNK kinases maintain ion homeostasis by controlling ion transporters and channels; for example, Na^+^-coupled Cl^-^ importers (NCC, NKCC1, and NKCC2) and K^+^-coupled Cl^-^ exporters (KCC1–KCC4) are essential for maintaining blood pressure and renal and nervous function^45^. Thus, intense efforts in the development of WNK inhibitors have been devoted to treating cardiovascular and renal diseases. WNKs have high sequence homology in their catalytic domains^39^ and share some downstream effectors. However, individual members have diverse targets and functions^46^. *WNK3* levels are low in normal lung tissues (https://gtexportal.org/home/gene/WNK3), but its expression is increased in some lung adenocarcinoma patients; approximately 6% of lung cancer patients have FPKM values > 1 (https://www.proteinatlas.org^24^). As shown in Fig. 6g, lung cancer patients with high *WNK3* expression showed shorter survival, and high *WNK3* expression could be a poor prognostic marker of cancer. Nevertheless, the relevance of WNK3 function in tumorigenesis is not yet well understood. WNK3 has a prosurvival role in HeLa cells by delaying the onset of apoptosis^31^ and facilitates glioma cell migration and invasion by modulating cell volume through the regulation of the downstream effector NKCC1^47^. We first demonstrated that WNK3 transactivates PD-L1 expression through the action of JNK and c-JUN (Fig. 4c–h). c-JUN regulates PD-L1 by directly binding to its enhancer^48^. Although there is some evidence to show that activated ERK promotes a functional c-JUN/PD-L1 axis in melanoma cells^49^, further studies are needed to fully elucidate the molecular mechanisms underlying WNK3 regulation of JNK/c-JUN.

Typical functional genomics-based screenings to identify therapeutic targets in cancer are being conducted in a neoplastic cell context^5–8^. This approach often leads to an inconsistent outcome in an in vivo setting where systemic perturbation is inevitable. In particular, the consequences of target perturbation in immune cells can directly affect tumor growth and survival. In our study, SIK3 inhibition robustly decreased neoplastic PD-L1 expression (Fig. 1e, f and Supplementary Fig. 1h, i). However, it also had a pleiotropic inhibitory effect on immune cells, resulting in decreased granzyme B release, making the cancer cells resistant to immune attack in the coculture condition (Supplementary Fig. 2k, l). The results from previous studies corroborate our finding that SIK inhibition suppresses immune cell secretion of proinflammatory cytokines, such as IL-6, IL-12, and TNF-α, while enhancing the secretion of anti-inflammatory cytokines, such as IL-10^50^. This is a good example demonstrating the limitation of functional genomic screenings in the neoplastic context to discover immuno-oncology targets. In contrast, both genetic depletion and chemical inhibition of WNK3 enhanced the cytolytic activity of effector T cells, as shown by the increased production of immune-activating cytokines and cytolytic enzymes in CD8^+^ T cells (Fig. 2c–f and Supplementary Fig. 2g, h), suggesting that WNK3 inhibition acts in a synergistic manner for a sustained antitumor effect.

Inferred by the fact that inhibition of the K^+^ channel ROMK1 is mediated by WNK3’s carboxyl terminus, not by WNK3’s kinase activity^51^, WNK3 could function in either a kinase activity-dependent or activity-independent manner similar to other WNK family proteins. Thus, we validated that WNK3 kinase activity is responsible for PD-L1 regulation by showing that the kinase-dead mutant WNK3-K159M failed to promote PD-L1 expression. However, unfortunately, as no WNK3-selective inhibitor is currently available, which is a limitation of this study, we instead used the potent pan-WNK inhibitor WNK463 for in vivo validation of WNK3 function. WNK463 inhibits WNK1, WNK2, WNK3, and WNK4 with IC50 values of 5, 1, 6, and 9 nM, respectively^52^. The notion that WNK1 knockout mice are embryonic lethal^53^, which, together with the fact that the PD-L1 regulatory axis is confined to WNK3, raises some toxicity concerns for WNK463. Indeed, a high exposure of WNK463 produces an unacceptable preclinical safety profile^52^. Unlike the case of *Wnk1*, *Wnk3* knockout mice were viable and normal except for their low blood pressure in mice fed a low-salt diet^54^. Except for this, other knockout phenotypes associated with systemic abnormalities have not been reported. Therefore, WNK3-selective kinase inhibitors warrant further development and investigation. Such inhibitors might have clinical utility against cancers responsive to anti-PD-1 or anti-PD-L1 therapies, including tumors with microsatellite instability and high tumor mutation burden. They may also be used as a combination therapy with immune checkpoint blockades such as anti-CTLA4, anti-LAG3 and anti-TIGIT in addition to anti-PD-1 investigated in this study to achieve long-term and more durable effects against tumor growth and progression.

## Supplementary information

Supplemental Information
