# Supplementary material for: WNK3 inhibition elicits antitumor immunity by suppressing PD-L1 expression on tumor cells and activating T-cell function
Source: Exp Mol Med. 2022 Nov 10;54(11):1913–26. doi: 10.1038/s12276-022-00876-z (PMC9722663; doi:10.1038/s12276-022-00876-z)
Supplement: Supplementary file 1 — Supplemental Information [file 12276_2022_876_MOESM1_ESM.pdf]

SUPPLEMENTAL INFORMATION

WNK3 inhibition elicits anti-tumor immunity by suppressing PD-L1 expression on tumor cells and activating T cell function

Hyun Ju Yoon, Gi-Cheon Kim, Sejin Oh, Hakhyun Kim, Yong Keon Kim, Yunji Lee, Min Seo Kim, Gino Kwon, Yeon-Su Ok, Ho-Keun Kwon*, Hyun Seok Kim*

*Email: hsfkim@yuhs.ac (Hyun Seok Kim); hk@yuhs.ac (Ho-Keun Kwon)

This file includes:

Supplementary Materials and Methods

Supplementary Fig. 1-5

Supplementary Table 1-6

Supplementary References

SUPPLEMENTARY MATERIALS AND METHODS

Gene set enrichment analysis and visualization

Integrated gene-set enrichment analysis was performed on the 73 hit genes obtained by screening (46 PD-L1 enhancers and 27 PD-L1 suppressors) using the Metascape webtool[1] (Gene Ontology, MSigDB, KEGG, Reactome, Hallmark gene sets) (https://metascape.org, v.3.5). Entire genes targeted by the pooled shRNAs were used as a background with default parameters (*p*-value cutoff, 0.01; minimum overlap, 3; minimum enrichment factor, 1.5). Significantly enriched gene sets (hypergeometric $p < 0.001$) were visualized using the Enrichment Map in Cytoscape (v.3.8.2)[2].

Genetic and chemical perturbation

Transduction: shRNA plasmids for lentiviral packaging were obtained from Sigma-Aldrich (St. Louis, MO, USA) as follows: WNK1 (TRCN0000219719); WNK2 (TRCN0000194773); WNK3 (TRCN0000219674, TRCN0000001531, TRCN0000195276, TRCN0000196566); HSP90AA1 (TRCN0000315007, TRCN0000001028); CA3 (TRCN0000151024, TRCN0000184026); CAST (TRCN0000073638, TRCN0000073642); PFKFB4 (TRCN0000037764, TRCN0000199612); SLC7A7 (TRCN0000043033, TRCN0000043037); HDAC3 (TRCN0000004824, TRCN0000196925); HSP90B1 (TRCN0000029427, TRCN0000276250); SIK3 (TRCN0000194845, TRCN0000037450); PAN3 (TRCN0000049805, TRCN0000049807); CTRL (TRCN0000003652, TRCN0000003651); SMAD4 (TRCN0000010321, TRCN0000040031); OSGEPL1 (TRCN0000047052, TRCN0000047050); PD-L1 (TRCN0000056913, used for H460). pGIPZ-shPD-L1 (#RHS4430-200253051, used for H2009) was purchased from Horizon Discovery (Waterbeach, UK). pGIPZ-PD-L1 WT[3] was obtained as a gift from Mien-Chie Hung (#121486, Addgene, Watertown, MA, USA, used for H2009). For the lentiviral construct encoding human WNK3, WNK3-Myc-DDK (#RC220755) was subcloned into pLVX vector (Clontech Laboratories, Mountain View, CA, USA). Lentivirus was produced by co-transfecting 1.3×10^6 HEK 293FT cells with 1 μg of the plasmid DNA, 0.75 μg psPAX2, and 0.25 μg pMD2.G (Addgene) and was added to 5×10^5 target cells seeded in 60-mm dishes. Then, transduced cells were selected with 1 μg/mL (H2009) or 2 μg/mL (H460) puromycin.

cDNA transfection: 4×10^5 H2009 cells or 8×10^5 H460 cells seeded in 6-well plates were

transfected with 2 μg (H2009) or 4 μg (H460) of plasmids using Lipofectamine 2000 (Invitrogen, Carlsbad, CA, USA) and then harvested 72 hours after transfection. The Myc-DDK-tagged plasmids encoding human WNK1 (#RC218208), WNK2 (#RC212364), WNK3 (#RC220755), and WNK4 (#RC223269) were purchased from OriGene (Rockville, MD, USA). The kinase-dead mutant construct of WNK3 (WNK3 K159M) was generated using a Q5 Site-Directed Mutagenesis Kit (#E0554S, New England Biolabs, Ipswich, MA, USA) following the manufacturer's guidelines. Primer sequences used were as follows, WNK3 K159M: forward, 5'-AGGAGCATTTATGACAGTATATAAAGG-3', reverse, 5'-CTTCCTAGTTCTATGTCAAATTTC-3'.

siRNA transfection: Cells (2×10^5) were treated with 50 nM siRNA mixed with RNAiMAX (Invitrogen) in each well of 6-well plates and were subjected to different experiments 72 hours after transfection. siRNA oligonucleotides were custom synthesized (Genolution, Seoul, Korea) with the following sequences, human WNK4: 5′-GAUUGCAGCUGCCAUGGUA-3′, mouse WNK3: 5′-GCCTCACGTTTGTCAGTAT-3′, 5′-ATACTGACAAACGTGAGGC-3′, negative control: 5′-GCAGGACCAGGCCAUAUGA-3′.

Chemical compounds: WNK463 (#CD00005886, Crysdot, Bel Air, MD, USA), dasatinib (#S1021, Selleckchem, Houston, TX, USA), YKL 06-062 (#AOB37242, AOBIOUS, Gloucester, MA, USA), YKL-05-099 (#HY-101147, MedChemExpress, Monmouth Junction, NJ, USA), PU-H71 (#NSC 750424, Selleckchem), CH5138303 (#S7340, Selleckchem), RGFP966 (#S7229, Selleckchem), acetazolamide (#A6011, Sigma-Aldrich), 5MPN (#S656801, Sigma-Aldrich), JNK-IN-8 (#18096, Cayman Chemical, Ann Arbor, MI, USA), and AEBSF HCl (#A8456, Sigma-Aldrich) were used as described in the figure legends.

qRT-PCR assay

RT-PCR-generated cDNA templates were mixed with probes, and TaqMan Fast Advanced Master Mix (Thermo Fisher Scientific, Waltham, MA, USA) and run on a StepOne Plus real-time PCR system (Applied Biosystems, Waltham, MA, USA). For SYBR Green assay, reactions were performed with custom synthesized primers and QuantiNova SYBR Green PCR Kit (QIAGEN, Germantown, MD, USA). Probes for TaqMan assay were as follows, WNK3 (Hs00908643_m1), HSP90AA1 (Hs00743767_sH), CA3 (Hs00193123_m1), CAST (Hs00156280_m1), PFKFB4 (Hs00894603_m1), SLC7A7 (Hs00909952_m1), HDAC3 (Hs00187320_m1), HSP90B1 (Hs00427665_g1), SIK3 (Hs00228549_m1), PAN3 (Hs01107000_m1), CTRL (Hs00157187_m1), SMAD4 (Hs00929647_m1), OSGEPL1

(Hs01088658_g1), PD-L1 (Hs00204257_m1) and 18S (Hs99999901_s1). Primer sequences for SYBR Green assay were provided as Supplementary Table 6. Human 18S ribosomal RNA or mouse β-actin was used as an internal control.

Flow cytometric analysis of WNK463-treated or *Wnk3*-knockout mouse CD4⁺ and CD8⁺ T cells

Spleen specimens were excised from mice and prepared into single cell suspensions. $CD4^+$ and $CD8^+$ T cells were enriched using EasySep Mouse Naïve $CD4^+$ T cell isolation kits (#19765, STEMCELL Technologies, Vancouver, BC, Canada) with CD19 (6D5), B220 (RA3-6B2), CD11b (M1/70), CD11c (N418), and NK1.1 (PK136) biotinylated antibodies (BioLegend, San Diego, CA, USA). Naive $CD4^+$ and $CD8^+$ T cells were sorted using an MA900 sorter (SONY, San Jose, CA, USA). Cells were stimulated with plate-bounded anti-mouse CD3 (#BE0001-1, BioXCell, Lebanon, NH, USA)/anti-mouse CD28 (#BE0015-1, BioXCell) for 24 hours. The predesigned sgRNA for targeting mouse *Wnk3* gene was synthesized from Integrated DNA Technologies (IDT; Coralville, IA, USA). Pre-designed sgRNA sequences were as follows: negative control: 5'-mA*mC*mG* rArUrU rCrCrU rArArG rArUrG rCrUrU rGrCrG rUrUrU rUrArG rArGrC rUrArG rArArA rUrArG rCrArA rGrUrU rArArA rArUrA rArGrG rCrUrA rGrUrC rCrGrU rUrArU rCrArA rCrUrU rGrArA rArArA rGrUrG rGrCrA rCrCrG rArGrU rCrGrG rUrGrC mU*mU*mU* rU-3', *Wnk3*: 5'-mU*mA*mG* rUrUrC rGrArU rUrCrU rArUrG rArUrU rCrArG rUrUrU rUrArG rArGrC rUrArG rArArA rUrArG rCrArA rGrUrU rArArA rArUrA rArGrG rCrUrA rGrUrC rCrGrU rUrArU rCrArA rCrUrU rGrArA rArArA rGrUrG rGrCrA rCrCrG rArGrU rCrGrG rUrGrC mU*mU*mU* rU-3'. To form the ribonucleoprotein, Alt-R CRISPR-Cas9 guide RNA and Alt-R S.p. HiFi Cas9 Nuclease V3 (#1081061, IDT) were mixed and transfected into activated $CD4^+$ and $CD8^+$ T cells using Amaxa P3 primary cell 4D-nucleofector X kit (Lonza, Basel, Switzerland) with application of the electroporation program DN-100. Recovered cells were cultured for 72 hours prior to flow cytometric analysis.

To quantify PERFORIN and GRANZYME B production, $CD4^+$ and $CD8^+$ T cells ($2x10^5$) were treated with 1μM WNK463 or DMSO for 6 hours and stimulated with Dynabeads Mouse T-Activator CD3/CD28 (#11452D, Invitrogen) for 36 hours. BD GolgiStop (BD Biosciences, Franklin Lakes, NJ, USA) was added for the last 6 hours of the stimulation. *Wnk3*-knockout $CD4^+$ and $CD8^+$ T cells ($2x10^5$) were stimulated with Dynabeads Mouse T-Activator CD3/CD28 (Invitrogen) for 36 hours. BD GolgiStop (BD Biosciences) was added

for the last 6 hours of the stimulation. After stimulation, WNK463-treated and *Wnk3*-knockout CD4$^+$ and CD8$^+$ T cells were washed, and surface molecules were stained with anti-CD4 (GK1.5, BioLegend) and anti-CD8 (53-6.7, BioLegend). Cells were then fixed with eBioscience/Invitrogen Intracellular (IC) Fixation Buffer (Invitrogen), washed with 1x Permeabilization Buffer (Invitrogen) and stained with antibodies for anti-PERFORIN (S16009A, BioLegend) and anti-GRANZYME B (QA16A02, BioLegend).

To quantify phospho-AKT and phospho-S6 proteins, WNK463-treated or *Wnk3*-knockout mouse CD4$^+$ and CD8$^+$ T cells (2x10^5) were stimulated with Dynabeads Mouse T-Activator CD3/CD28 (Invitrogen) for 5 min. After stimulation, cells were immediately fixed by adding 20 volumes of pre-warmed 1x BD phosflow Lyse/Fix Buffer (#558049, BD Biosciences) and permeabilized with BD phosflow Perm/Wash buffer (#557885, BD Biosciences). Then, cells were stained with anti-phospho-AKT (Ser473) (#9271, Cell Signaling Technology, Danvers, MA, USA) and anti-phospho-S6 Ribosomal protein (Ser240/244) (#5364, Cell Signaling Technology) antibody, followed by PE Donkey anti-rabbit IgG (minimal x-reactivity) antibody (#406421, BioLegend). Cell acquisition was performed on BD FACSCelesta cell analyzer (BD Biosciences), and data were analyzed using FlowJo software.

Immunoblot analysis

Cells were washed with PBS and lysed in RIPA lysis buffer (Sigma-Aldrich) containing protease and phosphatase inhibitor cocktail (GenDEPOT, Katy, TX, USA). Protein concentrations were measured with Bradford reagent (Bio-Rad, Hercules, CA, USA). Equal amounts of protein were separated on 4-15% Mini-PROTEAN TGX Precast Gels (Bio-Rad). Anti-PD-L1 (#13684S), anti-p-JNK (#4668S), anti-JNK (#9252S), anti-p-c-Jun (#3270S), anti-p-ERK1/2 (#4370S), anti-ERK1/2 (#4348S), anti-p-p38 (#4631S), anti-p38 (#9212S), anti-p-AKT (#9271S), anti-AKT (#4691S), anti-p-P65 (#3033S), anti-P65 (#8242S), anti-p-STAT3 (#9145S), anti-STAT3 (#9139S), anti-HSP90 (#4877) and anti-flag(DYKDDDDK) (#2368S) antibodies were purchased from Cell Signaling Technology. Anti-PD-L1 (mouse specific, #ab213480, Abcam, Cambridge, MA, USA), and anti-β-actin (#sc-47778, Santa Cruz Biotechnology, Dallas, TX, USA) antibodies were purchased. Peroxidase AffiniPure Goat anti-rabbit IgG (#111-035-144) and anti-mouse IgG (#115-035-003, Jackson ImmunoResearch, West Grove, RA, USA) were used as secondary antibodies. Primary antibodies were diluted 1:1000 and secondary antibodies were used at 1:5000.

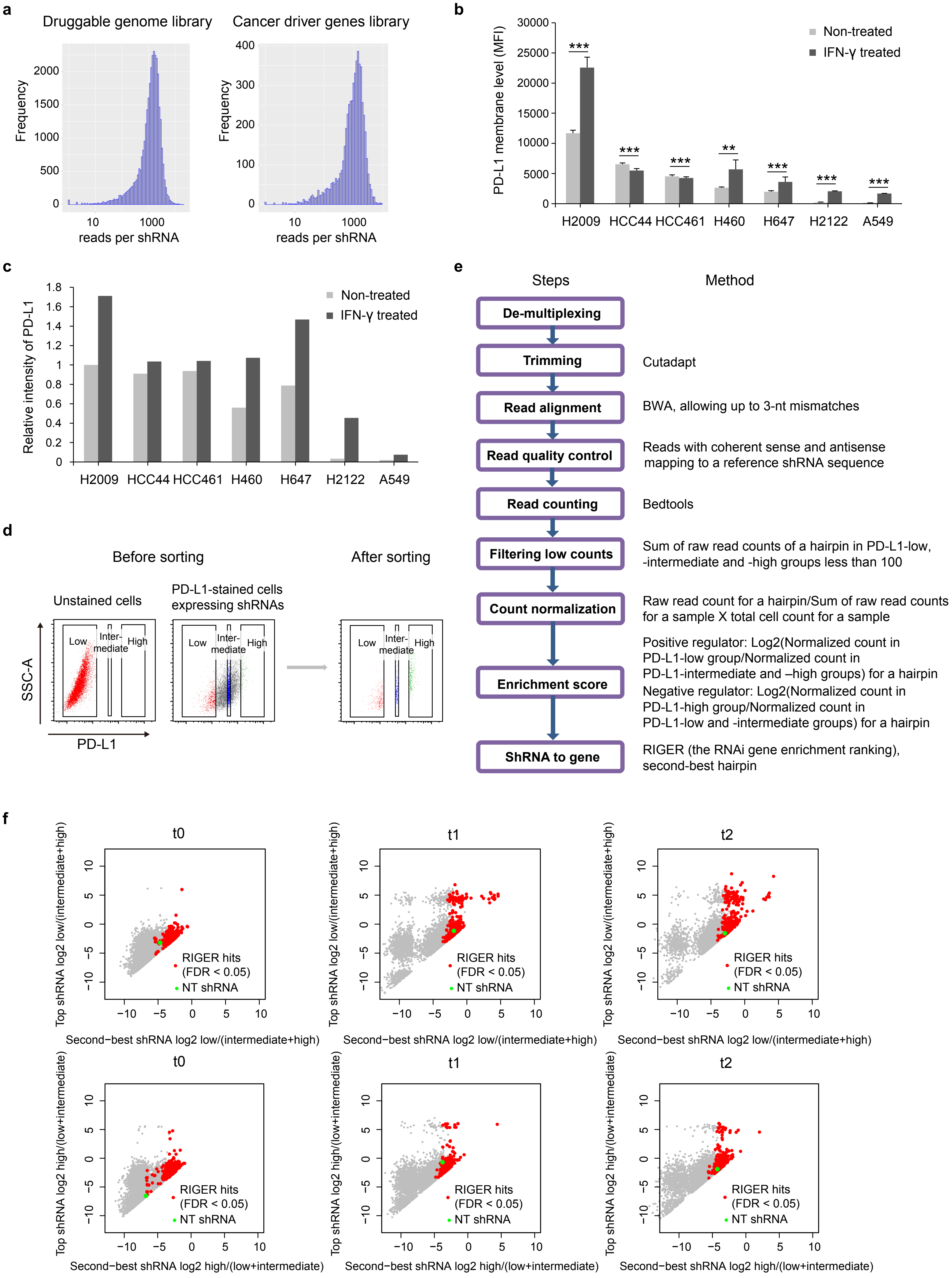

a
Druggable genome library
Cancer driver genes library
Frequency
reads per shRNA
b
PD-L1 membrane level (MFI)
Non-treated
IFN-γ treated
H2009 HCC44 HCC461 H460 H647 H2122 A549
c
Relative intensity of PD-L1
Non-treated
IFN-γ treated
H2009 HCC44 HCC461 H460 H647 H2122 A549
d
Before sorting
After sorting
Unstained cells
PD-L1-stained cells expressing shRNAs
Low
Inter-mediate
High
SSC-A
PD-L1
e
Steps
Method
De-multiplexing
Trimming
Cutadapt
Read alignment
BWA, allowing up to 3-nt mismatches
Read quality control
Reads with coherent sense and antisense mapping to a reference shRNA sequence
Read counting
Bedtools
Filtering low counts
Sum of raw read counts of a hairpin in PD-L1-low, -intermediate and -high groups less than 100
Count normalization
Raw read count for a hairpin/Sum of raw read counts for a sample X total cell count for a sample
Enrichment score
Positive regulator: Log2(Normalized count in PD-L1-low group/Normalized count in PD-L1-intermediate and –high groups) for a hairpin
Negative regulator: Log2(Normalized count in PD-L1-high group/Normalized count in PD-L1-low and -intermediate groups) for a hairpin
ShRNA to gene
RIGER (the RNAi gene enrichment ranking), second-best hairpin
f
t0
t1
t2
Top shRNA log2 low/(intermediate+high)
Second−best shRNA log2 low/(intermediate+high)
Top shRNA log2 high/(low+intermediate)
Second−best shRNA log2 high/(low+intermediate)
RIGER hits (FDR < 0.05)
NT shRNA

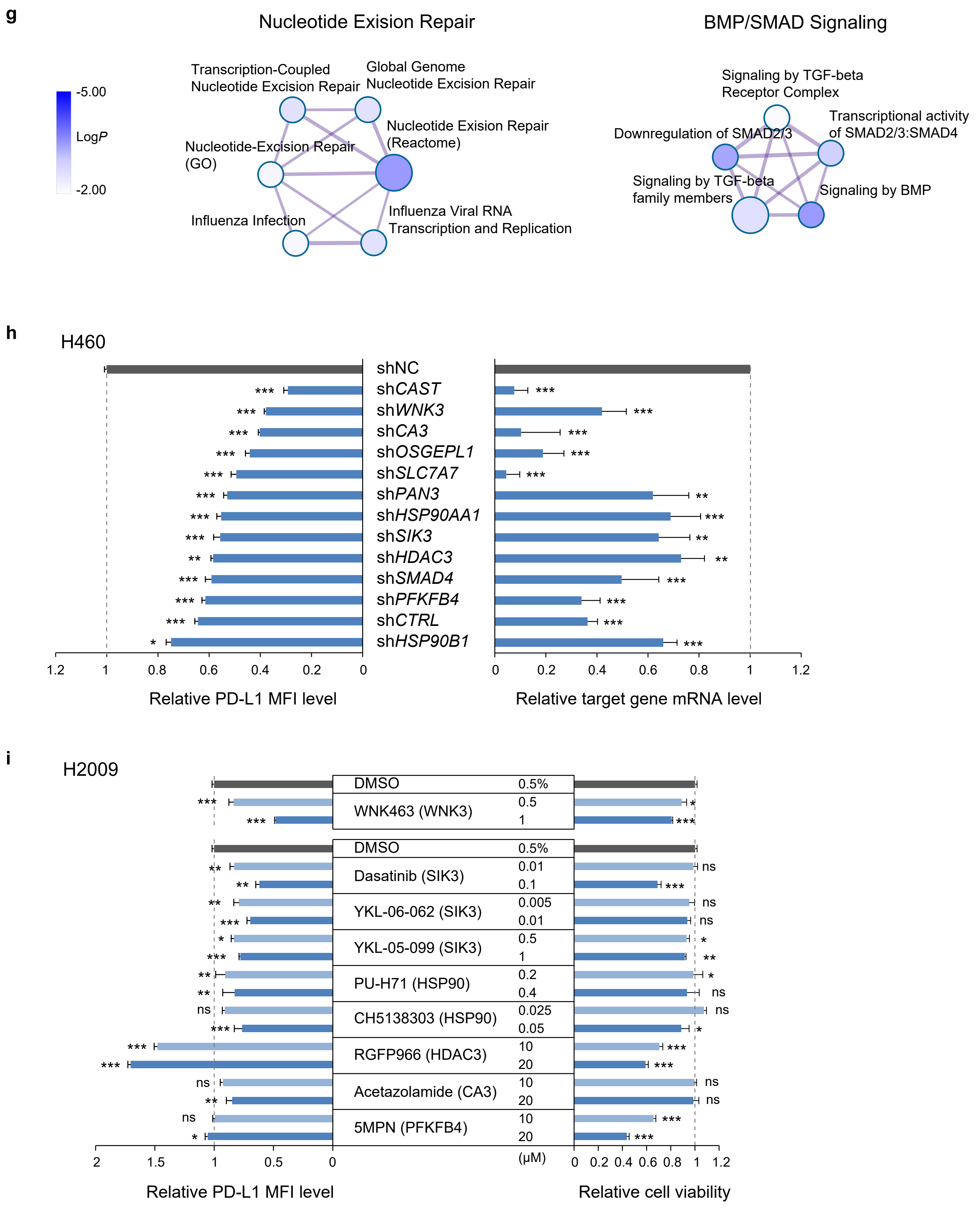

g
Nucleotide Exision Repair
BMP/SMAD Signaling
-5.00
LogP
-2.00
Transcription-Coupled Nucleotide Excision Repair
Global Genome Nucleotide Excision Repair
Nucleotide Exision Repair (Reactome)
Nucleotide-Excision Repair (GO)
Influenza Infection
Influenza Viral RNA Transcription and Replication
Signaling by TGF-beta Receptor Complex
Transcriptional activity of SMAD2/3:SMAD4
Downregulation of SMAD2/3
Signaling by TGF-beta family members
Signaling by BMP
h
H460
shNC
shCAST
shWNK3
shCA3
shOSGEPL1
shSLC7A7
shPAN3
shHSP90AA1
shSIK3
shHDAC3
shSMAD4
shPFKFB4
shCTRL
shHSP90B1
Relative PD-L1 MFI level
Relative target gene mRNA level
i
H2009
DMSO 0.5%
WNK463 (WNK3) 0.5 1
DMSO 0.5%
Dasatinib (SIK3) 0.01 0.1
YKL-06-062 (SIK3) 0.005 0.01
YKL-05-099 (SIK3) 0.5 1
PU-H71 (HSP90) 0.2 0.4
CH5138303 (HSP90) 0.025 0.05
RGFP966 (HDAC3) 10 20
Acetazolamide (CA3) 10 20
5MPN (PFKFB4) 10 20
(μM)
Relative PD-L1 MFI level
Relative cell viability

Supplementary Fig. 1 shRNA screening of druggable genes and cancer drivers that regulate PD-L1 expression. a Histograms of shRNA abundances targeting druggable genes (N=5,069; left) and cancer driver genes (N=800; right). **b** Bar plots represent the quantitative analysis of the flow cytometry data in Fig. 1**a**. Error bars indicate ± standard deviation (N=4). ** p <0.01, *** p <0.001; Statistical significance was calculated using two-sided unpaired Student's t-test. **c** Bar plots represent the quantitative densitometric analysis of the immunoblots in Fig. 1**b**. **d** Distribution of cells at t1 before and after FACS sorting by PD-L1 expression. The PD-L1-low population was defined and gated based on unstained cells expressing control shRNAs. The PD-L1-intermediate population expressing intermediate levels of PD-L1 (13-15% of total) was defined and gated by cells expressing control shRNAs. The PD-L1-high population represents 1-2% of cells with the highest PD-L1 expression. **e** Workflow for the highly parallel amplicon sequencing-based shRNA deconvolution process. **f** Identification of hit genes by RIGER analysis. Scatter plots of genes showing the best (y-axis) and the second-best corresponding shRNAs (x-axis) enrichment scores. Enrichments in the PD-L1-low and PD-L1-high populations are shown on the top and the bottom, respectively. Hit genes by RIGER analysis (FDR<0.05) are shown as red dots. NT shRNA: non-targeting shRNA. **g** Gene-set enrichment analysis of the hit genes. Significantly enriched (p < 0.001) gene sets are shown with an enrichment map with default parameters in which a node represents an individual gene set, and an edge represents the overlap between gene sets. Node size is proportional to the number of genes in the gene set. **h** Genetic validation of positive PD-L1 regulators. H460 cells were transduced with the best PD-L1 depleting shRNAs of the 13 validated genes in Fig. 1**e** (N=3). Error bars indicate ± standard deviation (N=3). * p <0.05, ** p <0.01, *** p <0.001; Two-sided unpaired Student's t-test. **i** Chemical validation of positive PD-L1 regulators. Flow cytometry analysis of PD-L1 (left) and cell viability assay (right) of H2009 cells after treatment with WNK463 (for 9 days), dasatinib, YKL-06-062, YKL-05-099, PU-H71, CH5138303, RGFP966, acetazolamide, 5MPN (for 5 days) or the equivalent concentration of DMSO (vehicle control). Error bars indicate ± standard deviation (N=3). * p <0.05, ** p <0.01, *** p <0.001, not significant (ns) p >= 0.05; Two-sided unpaired Student's t-test.

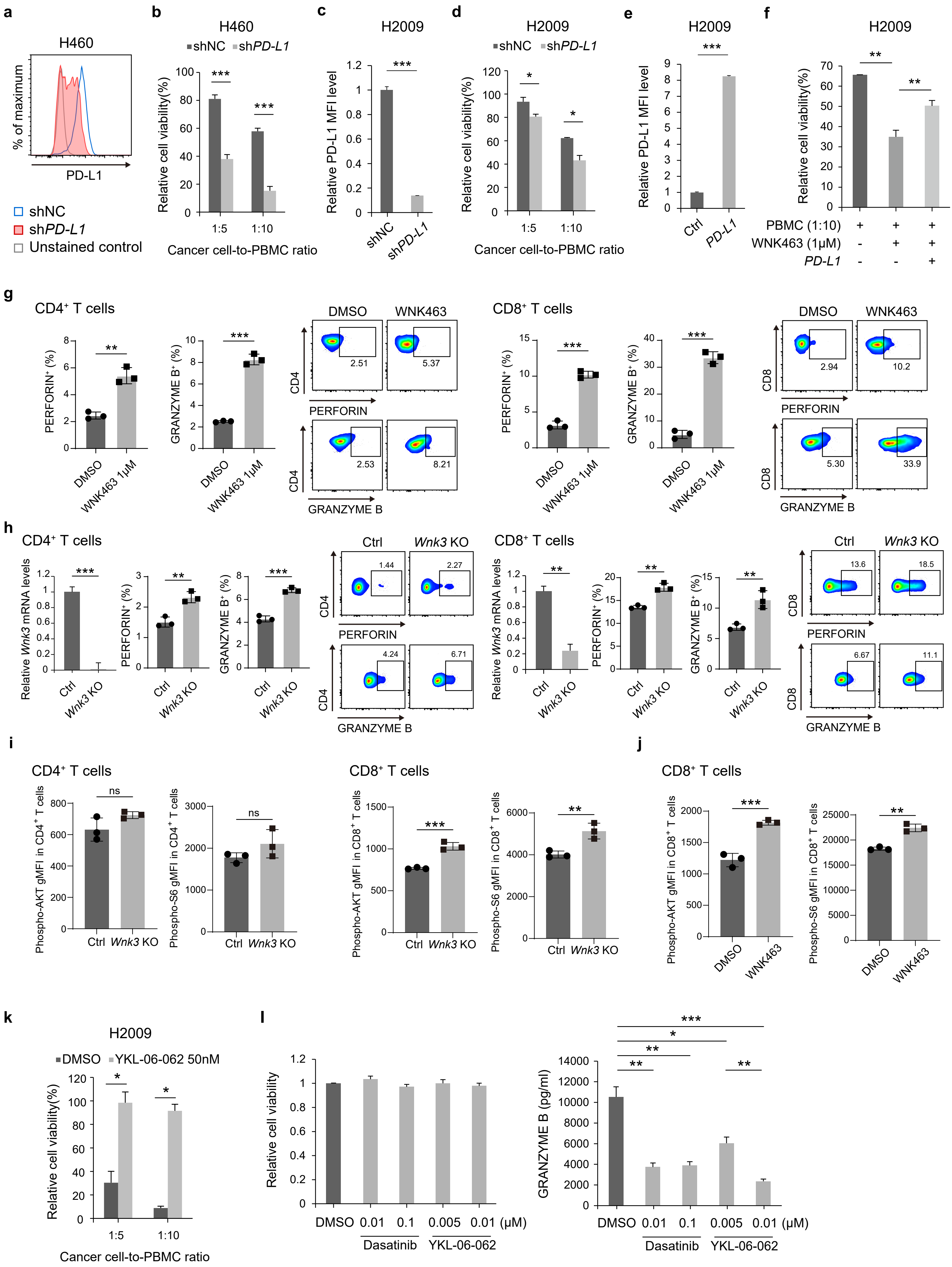

a
H460
% of maximum
PD-L1
shNC
shPD-L1
Unstained control
b
H460
shNC shPD-L1
Relative cell viability(%)
Cancer cell-to-PBMC ratio
c
H2009
Relative PD-L1 MFI level
shNC
shPD-L1
d
H2009
shNC shPD-L1
Relative cell viability(%)
Cancer cell-to-PBMC ratio
e
H2009
Relative PD-L1 MFI level
Ctrl
PD-L1
f
H2009
Relative cell viability(%)
PBMC (1:10)
WNK463 (1μM)
PD-L1
g
CD4+ T cells
PERFORIN+ (%)
GRANZYME B+ (%)
DMSO
WNK463 1μM
WNK463
CD4
PERFORIN
GRANZYME B
CD8+ T cells
CD8
h
CD4+ T cells
Relative Wnk3 mRNA levels
Ctrl
Wnk3 KO
CD8+ T cells
i
CD4+ T cells
Phospho-AKT gMFI in CD4+ T cells
Phospho-S6 gMFI in CD4+ T cells
CD8+ T cells
Phospho-AKT gMFI in CD8+ T cells
Phospho-S6 gMFI in CD8+ T cells
j
CD8+ T cells
k
H2009
DMSO YKL-06-062 50nM
Relative cell viability(%)
Cancer cell-to-PBMC ratio
l
Relative cell viability
GRANZYME B (pg/ml)
Dasatinib
YKL-06-062
(μM)

Supplementary Fig. 2 Genetic and chemical validation of the immune-modulatory effect of WNK3 and SIK3 in an *in vitro* co-culture condition. **a** Flow cytometry analysis of PD-L1 on H460 cells transduced with sh*PD-L1* or negative control (shNC). **b** Relative viability of H460 cells from **a** after 72 hours of co-culture with activated PBMCs (N=3). **c** Flow cytometry analysis of PD-L1 on H2009 cells transduced with sh*PD-L1* or negative control (shNC). **d** Relative viability of H2009 cells from **c** after 72 hours of co-culture with activated PBMCs (N=3). **e** Flow cytometry analysis of PD-L1 on H2009 cells transduced with lentiviral vectors expressing *PD-L1* transgene or with non-targeting control vectors. **f** Relative viability of H2009 cells from **e** after 48 hours of co-culture with activated PBMCs with 1 μM WNK463 or DMSO (vehicle). H2009 cells were pretreated with 1 μM WNK463 or DMSO for 9 days (N=3). **g** PERFORIN and GRANZYME B production in mouse $CD4^+$ T cells (left) or $CD8^+$ T cells (right) after 6 hours of treatment with 1 μM WNK463 or DMSO (vehicle) followed by 36 hours of TCR stimulation (N=3). **h, i** PERFORIN, GRANZYME B (**h**), phospho-AKT and phospho-S6 (**i**) levels in mouse $CD4^+$ T cells (left) or $CD8^+$ T cells (right) transfected with a ribonucleoprotein complex composed of sgRNA (sg*Wnk3* or non-targeting sgRNA (Ctrl)) and Cas9 protein after 36 hours (**h**) or 5min (**i**) of TCR stimulation (N=3). *Wnk3* depletion was measured by qRT-PCR (N=3). **j** Phospho-AKT and phospho-S6 levels in mouse $CD8^+$ T cells after 6 hours of treatment with 1 μM WNK463 or DMSO (vehicle) followed by 5 min of TCR stimulation (N=3). **k** Relative viability of H2009 cells after 96 hours of co-culture with activated PBMCs with 50 nM YKL-06-062 or DMSO (N=3). **l** Relative viability (left) and released GRANZYME B (right) of PBMCs after 72-hour treatment with the indicated pan-SIK inhibitors or DMSO (N=2). Statistical differences were determined by a two-sided unpaired Student's t-test (**b-k**) or an one-way ANOVA followed by a Tukey multiple comparison test (**l**). * p <0.05, ** p <0.01, *** p <0.001, not significant (ns) p >= 0.05. Error bars indicate ±standard deviation (**b-f**, **k-l**) or ±standard error of mean (**g-j**).

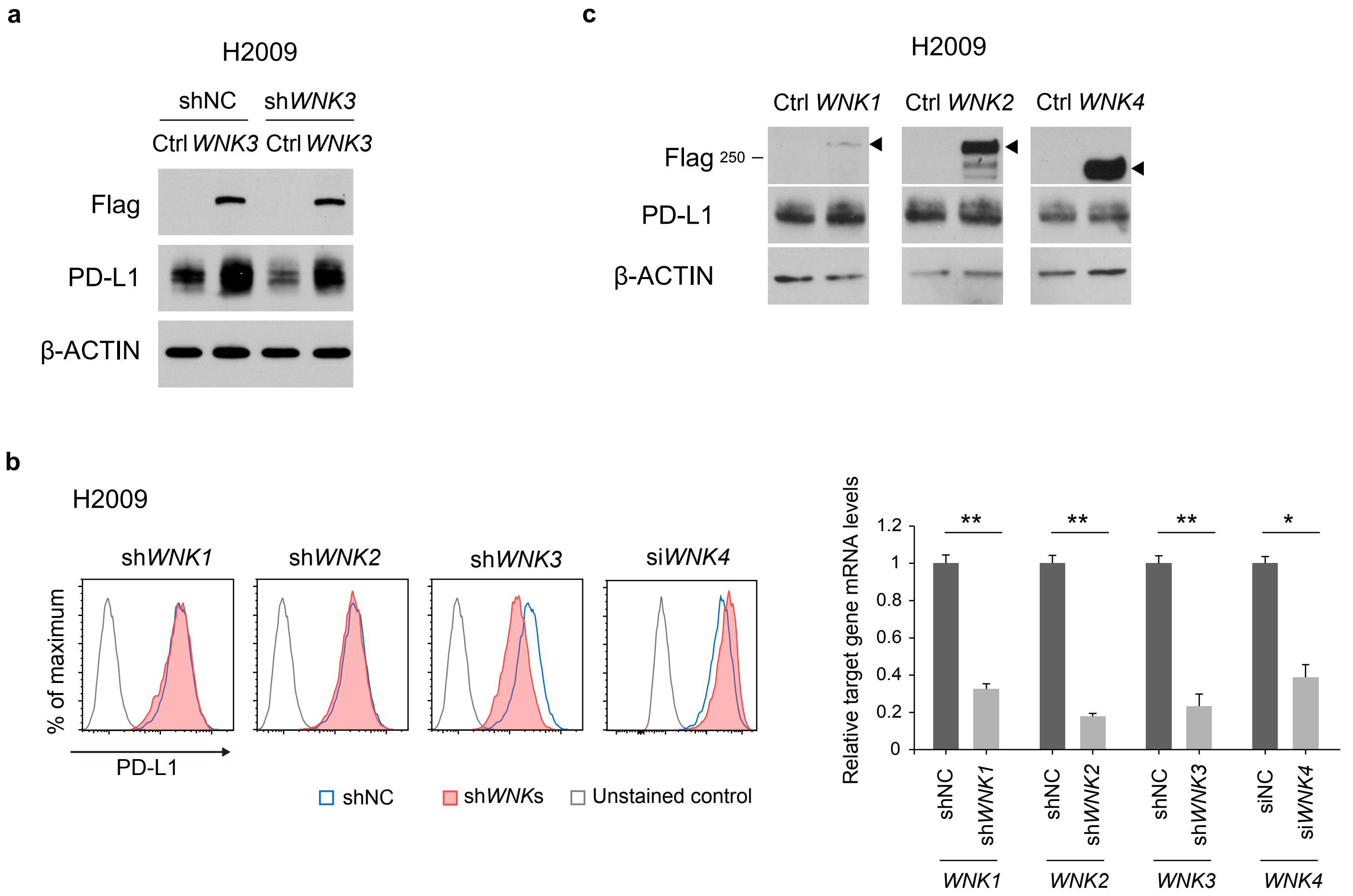

Supplementary Fig. 3 WNK3 is a transcriptional regulator of *PD-L1*. a Effect of *WNK3* transgene overexpression on the PD-L1 levels reduced by sh*WNK3* targeting endogenous *WNK3*. H2009 cells expressing sh*WNK3* targeting the 3' UTR region of the human *WNK3* gene were transfected with *WNK3* cDNA plasmids or with empty vectors. PD-L1 protein levels were determined by immunoblotting. β-actin was used as a loading control. **b** PD-L1 membrane levels in H2009 cell lines expressing indicated shRNAs targeting different WNK family proteins are examined by flow cytometry (left). Target gene knockdown efficiencies are shown in the bar plots (right). Error bars indicate ± standard deviation (N=2). * p <0.05, ** p <0.01; Statistical significance was calculated using two-sided unpaired Student's t-test. **c** Effect of overexpression of the indicated WNK family proteins on the PD-L1 levels in the H2009 cell line. Arrowheads indicate FLAG-fused proteins. β-actin was used as a loading control.

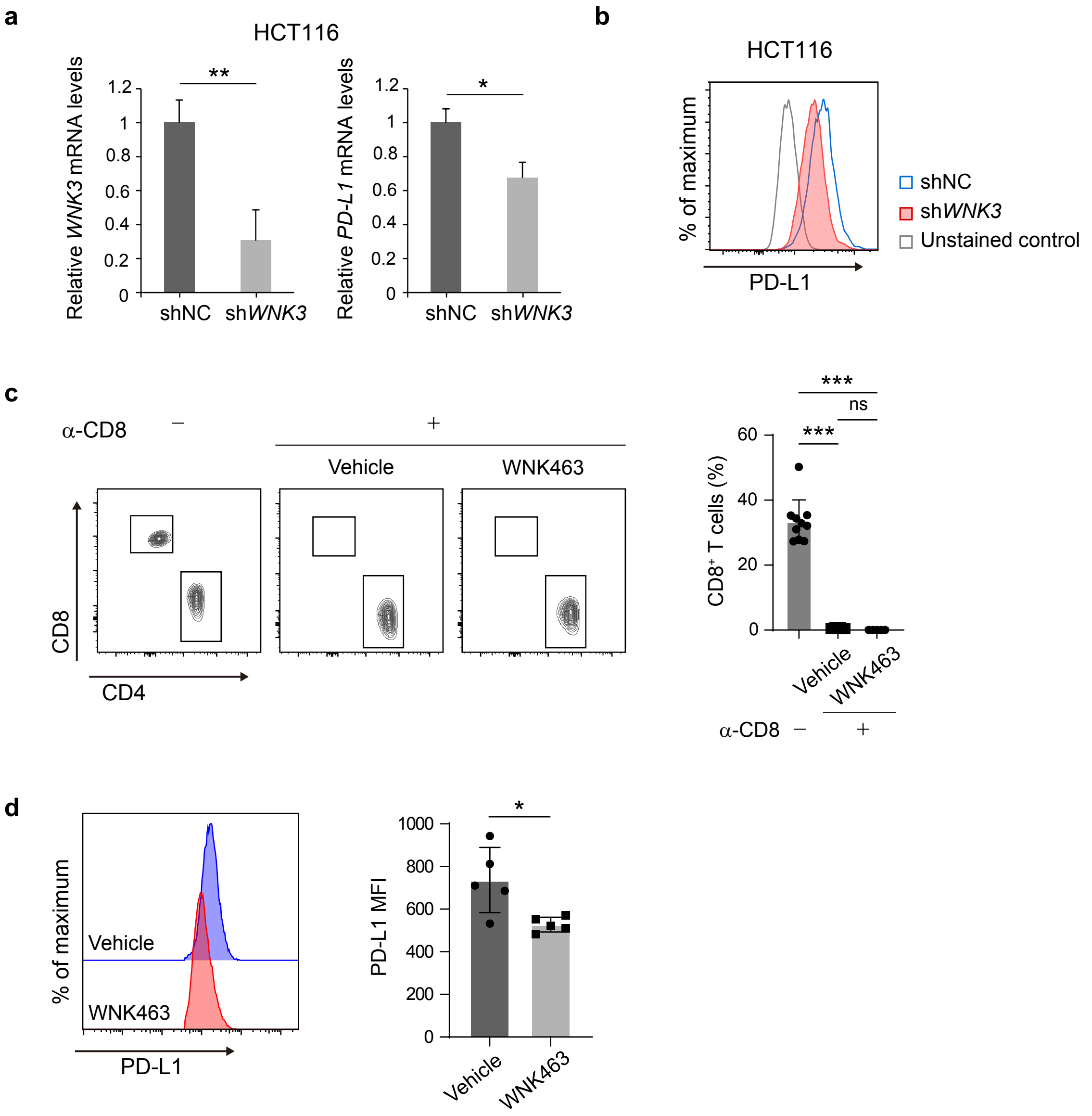

Supplementary Fig. 4 Genetic and chemical inhibition of WNK3 sensitizes PD-L1 dependent syngeneic tumors to anti-PD-1 therapy. a mRNA levels of *WNK3* (left) and *PD-L1* (right) in HCT116 cells expressing sh*WNK3* or non-targeting shRNA (shNC) were determined by qRT-PCR (N=3). **b** PD-L1 levels in HCT116 expressing sh*WNK3* or non-targeting shRNA were examined by flow cytometry. **c** $CD4^+$ and $CD8^+$ T cells extracted from non-treated (Fig. 5**f**, left) or anti-CD8 antibody-treated mice (Fig. 5**f**, right) were analyzed by flow cytometry. **d** PD-L1 levels in extracted MC38 tumor cells (Fig. 5**f**, right) were evaluated by flow cytometry. Cells were pre-gated on live $CD45^+TCRb^+$. Statistical significance was determined by a two sided unpaired Student's t-test (**a**, **d**) or an ordinary one-way ANOVA followed by a Tukey multiple comparison test with a single pooled variance (**c**). * p <0.05, ** p <0.01, *** p <0.001, not significant (ns) p >= 0.05. Error bars indicate ±standard deviation.

a

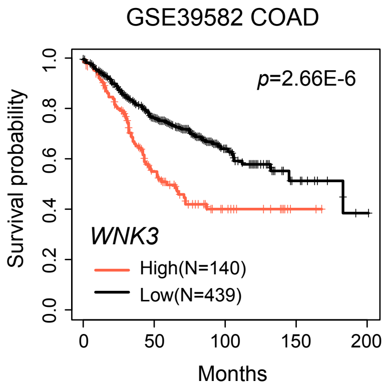

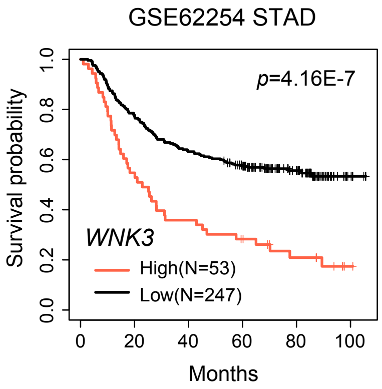

Supplementary Fig. 5 High *WNK3* expression is associated with poor overall survival in colorectal and gastric cancer patients. a Kaplan-Meier survival analysis of overall survival for colon (left) and gastric (right) cancer patients with high vs. low *WNK3* expression in the indicated cohorts. Patients were classified according to the optimal gene expression thresholds[4].

Supplementary Table 1. Druggable genes used in this study[5]

Data type	Source[ref]	Genes
**Druggable genes**	GO[6]	5815
	Russ & Lampel[7]	3026
	Hopkins & Groom[8]	2668
	dGene[9]	2256
	BaderLabGenes[10]	292
	FoundationOneGenes[11]	229
**Drug-gene interactions**	DrugBank[12]	2049
	PharmGKB[13]	594
	TTD[14]	477
	TEND[15]	430
	MyCancerGenome[16]	169
	TALC[17]	153
	ClearityFoundationClinicalTrial*	93
	MyCancerGenomeClinicalTrial[18]	78
	CancerCommons[19]	48
	ClearityFoundationBiomarkers*	34
	Totals (unique)	5069

* https://www.clearityfoundation.org (data is no longer publicly available from site)

Supplementary Table 2. Cancer driver genes used in this study

	Reference	Tumor types	Cancer driver genes
Pan-cancer studies			
-Somatic mutations			
**a**	Ciriello G. et al. Nat Genet 45, 1127-33 (2013)[20]	13	199
**b**	Kandoth C. et al. Nature 502, 333-9 (2013)[21]	13	127
**c**	Vogelstein, B. et al. Science 339, 1546–58 (2013)[22]	26	138
**d**	Chang M. T. et al. Nat Biotechnol 34, 155–163 (2016)[23]	41	275
-Somatic copy number alterations			
**e**	Zack TI. et al. Nat Genet 45, 1134-40 (2013)[24]	12	131
**f**	Beroukhim R. et al. Nature 463, 899-905 (2010) [25]	26	196
Gastric cancer studies			
-Somatic mutations			
**g**	Cancer Genome Atlas Research Network. Nature 513, 202-9 (2014)[26]	1	57
**h**	Cristescu R. et al. Nat Med 21, 449-56 (2015)[27]	1	17
	Totals (unique)		800

Supplementary Table 3. 73 candidate genes enhancing or suppressing PD-L1 expression

	Category	Gene symbols	Count
PD-L1 enhancer	Druggable gene	*AKR1C1, BMPR2, CA3, CAST, CTRL, EPHB1, GGT5, GLRX, GSTT2, HCK, HDAC3, HSP90AA1, HSP90B1, KCNB2, KSR1, METAP1, NDUFS3, OAZ2, OR4Q3, OR52E2, OR9K2, OSGEPL1, PFKFB4, PLAUR, PSKH1, SDHC, SERPINA10, SIK3, SLC25A17, SLC7A7, SLC9A3, TAS2R13, TAS2R40, TIGD1, UQCRC1, USP17, WNK3*	37
	Cancer driver gene	*ARIH1, BRD1, CDKN2C, MYH3, NDRG2, SMAD4*	6
	Druggable cancer driver gene	*ADAMTS20, CCDC105, PAN3*	3
PD-L1 suppressor	Druggable gene	*EWSR1, PSMD9, NT5C2, RIOK3, POLE4, APOE, POLR2C, COPS5, CD70, RPS6KA6, CPE, CRYBB1, FCGR3B, LDHC, CDKL3, ACBD4, BTNL3, TGM6, OR52L1, TRIM49L1*	20
	Cancer driver gene	*SMC3, SRSF2, UBE2D3, CHL1, IGFBP1, MYO18A*	6
	Druggable cancer driver gene	*PARP1*	1

Supplementary Table 4. List of chemical compounds that inhibit PD-L1 enhancers

PD-L1 enhancer (Gene symbol)	Compound name	Target specificity
WNK3	WNK463	Pan-WNK
SIK3	Dasatinib	Pan-SIK
	YKL-06-062	Pan-SIK
	YKL-05-099	Pan-SIK
HSP90AA1, HSP90B1	PU-H71	Pan-Hsp90
HSP90AA1	CH5138303	Hsp90a
HDAC3	RGFP966	HDAC3
CA3	Acetazolamide	Pan-CA
PFKFB4	5MPN	PFKFB4

Supplementary Table 5. Primer sequences for shRNA amplification

primer	sequence
**1st PCR forward**	5′-AATGGACTATCATATGCTTACCGTAACTTGAAAGTATTTCG-3′[28]
**1st PCR reverse**	5′-CTTTAGTTTGTATGTCTGTTGCTATTATGTCTACTATTCTTTCCC-3′[28]
**2nd PCR forward**	5′-AATGATACGGCGACCACCGAGATCTACACTCTTTCCCTACACGACGCTCTTCCGATCTNNNNNNNNNNTCTTGTGGAAAGGACGA-3′<br>5′-AATGATACGGCGACCACCGAGATCTACACTCTTTCCCTACACGACGCTCTTCCGATCTNNNNNNNNNNGATCTTGTGGAAAGGACGA-3′<br>5′-AATGATACGGCGACCACCGAGATCTACACTCTTTCCCTACACGACGCTCTTCCGATCTNNNNNNNNNNCGATCTTGTGGAAAGGACGA-3′<br>5′-AATGATACGGCGACCACCGAGATCTACACTCTTTCCCTACACGACGCTCTTCCGATCTNNNNNNNNNNACGATCTTGTGGAAAGGACGA-3′<br>5′-AATGATACGGCGACCACCGAGATCTACACTCTTTCCCTACACGACGCTCTTCCGATCTNNNNNNNNNNCTAGAATCTTGTGGAAAGGACGA-3′<br>5′-AATGATACGGCGACCACCGAGATCTACACTCTTTCCCTACACGACGCTCTTCCGATCTNNNNNNNNNNTGGACACATCTTGTGGAAAGGACGA-3′<br>5′-AATGATACGGCGACCACCGAGATCTACACTCTTTCCCTACACGACGCTCTTCCGATCTNNNNNNNNNNGTCGGCACATCTTGTGGAAAGGACGA-3′<br>5′-AATGATACGGCGACCACCGAGATCTACACTCTTTCCCTACACGACGCTCTTCCGATCTNNNNNNNNNNAACCAGCACATCTTGTGGAAAGGACGA-3′
**2nd PCR reverse**	5′-CAAGCAGAAGACGGCATACGAGATNNNNNNNNGTGACTGGAGTTCAGACGTGTGCTCTTCCGATCTTCTACTATTCTTTCCCCTGCACTGT-3′

Supplementary Table 6. Primer sequences for SYBR Green qRT-PCR assay

Gene	Primer Sequence
**Human PD-L1**	Forward, 5′-CAATGTGACCAGCACACTGAGAA-3′<br>Reverse, 5′-GGCATAATAAGATGGCTCCCAGAA-3′
**Human WNK1**	Forward, 5′- GCCGTCAGATCCTTAAAGGTC-3′<br>Reverse, 5′-CCAGTAGGGCCGGTGATAA-3′
**Human WNK2**	Forward, 5′-CGCTTCCTCAAGTTCGACATC-3′<br>Reverse, 5′-TGGACTCCCAGAAGTCGTAGA-3′
**Human WNK3**	Forward, 5′-ACTTCTCCTAGTGGCAGATTCC-3′<br>Reverse, 5′-GCAGCTCACACCAAGCAAC-3′
**Human WNK4**	Forward, 5′-CGATGGCCGATACCTCAAGTT-3′<br>Reverse, 5′-GTCGGTGTCTAGCCCTCGAT-3′
**Human 18S**	Forward, 5′-ACTCAACACGGGAAACCTCA-3′<br>Reverse, 5′-AACCAGACAAATCGCTCCAC-3′
**Mouse PD-L1**	Forward, 5’-TGCGGACTACAAGCGAATCACG-3’<br>Reverse, 5′- CTCAGCTTCTGGATAACCCTCG-3′
**Mouse WNK3**	Forward, 5′-GGTGGTCAGTCTTCAAACACAA-3′<br>Reverse, 5′-GTGAACATCCCCTTCTTACTGG-3′<br>Forward, 5′-ACTTCTCCTAGTGGCAGATTCC-3′ (in Supplementary Fig. 2h)<br>Reverse, 5′-GCAGCTCACACCAAGCAAC-3′ (in Supplementary Fig. 2h)
**Mouse β-actin**	Forward, 5′-GTGACGTTGACATCCGTAAAGA-3′<br>Reverse, 5′-GCCGGACTCATCGTACTCC-3′

SUPPLEMENTARY REFERENCES

1 Zhou, Y. *et al.* Metascape provides a biologist-oriented resource for the analysis of systems-level datasets. *Nat. Commun.* **10**, 1-10 (2019).

2 Shannon, P. *et al.* Cytoscape: a software environment for integrated models of biomolecular interaction networks. *Genome Res.* **13**, 2498-2504 (2003).

3 Li, C.-W. *et al.* Glycosylation and stabilization of programmed death ligand-1 suppresses T-cell activity. *Nat. Commun.* **7**, 12632 (2016).

4 Uhlen, M. *et al.* A pathology atlas of the human cancer transcriptome. *Science (1979)* **357** (2017).

5 Griffith, M. *et al.* DGIdb: mining the druggable genome. *Nat. Methods* **10**, 1209 (2013).

6 Ashburner, M. *et al.* Gene ontology: tool for the unification of biology. *Nat. Genet.* **25**, 25-29 (2000).

7 Russ, A. P. & Lampel, S. The druggable genome: an update. *Drug Discov. Today* **10**, 1607-1610 (2005).

8 Hopkins, A. L. & Groom, C. R. The druggable genome. *Nat. Rev. Drug Discov.* **1**, 727-730 (2002).

9 Kumar, R. D., Chang, L.-W., Ellis, M. J. & Bose, R. Prioritizing potentially druggable mutations with dGene: an annotation tool for cancer genome sequencing data. *PLoS One* **8**, e67980 (2013).

10 Edwards, A. M. *et al.* Too many roads not taken. *Nature* **470**, 163-165 (2011).

11 Wagle, N. *et al.* High-throughput detection of actionable genomic alterations in clinical tumor samples by targeted, massively parallel sequencing. *Cancer Discov.* **2**, 82-93 (2012).

12 Law, V. *et al.* DrugBank 4.0: shedding new light on drug metabolism. *Nucleic Acids Res.* **42**, D1091-D1097 (2014).

13 McDonagh, E. M., Whirl-Carrillo, M., Garten, Y., Altman, R. B. & Klein, T. E. From pharmacogenomic knowledge acquisition to clinical applications: the PharmGKB as a clinical pharmacogenomic biomarker resource. *Biomark. Med.* **5**, 795-806 (2011).

14 Zhu, F. *et al.* Update of TTD: therapeutic target database. *Nucleic Acids Res.* **38**, D787-D791 (2010).

15 Rask-Andersen, M., Almén, M. S. & Schiöth, H. B. Trends in the exploitation of novel drug targets. *Nat. Rev. Drug Discov.* **10**, 579-590 (2011).

16 Yeh, P. *et al.* DNA-Mutation Inventory to Refine and Enhance Cancer Treatment (DIRECT): a catalog of clinically relevant cancer mutations to enable genome-directed anticancer therapy. *Clin. Cancer Res.* **19**, 1894-1901 (2013).

17 Somaiah, N. & Simon, G. R. Molecular targeted agents and biologic therapies for lung cancer. *J. Thorac. Oncol.* **6**, S1758-S1785 (2011).

18 Jain, N. *et al.* The My Cancer Genome clinical trial data model and trial curation workflow. *J. Am. Med. Inform. Assoc.* **27**, 1057-1066 (2020).

19 Bingham, A. *Collaborative computational technologies for biomedical research*. Vol. 10 (John Wiley & Sons, 2011).
20 Ciriello, G. *et al.* Emerging landscape of oncogenic signatures across human cancers. *Nat. Genet.* **45**, 1127 (2013).
21 Kandoth, C. *et al.* Mutational landscape and significance across 12 major cancer types. *Nature* **502**, 333 (2013).
22 Vogelstein, B. *et al.* Cancer genome landscapes. *Science (1979)* **339**, 1546-1558 (2013).
23 Chang, M. T. *et al.* Identifying recurrent mutations in cancer reveals widespread lineage diversity and mutational specificity. *Nat. Biotechnol.* **34**, 155 (2016).
24 Zack, T. I. *et al.* Pan-cancer patterns of somatic copy number alteration. *Nat. Genet.* **45**, 1134 (2013).
25 Beroukhim, R. *et al.* The landscape of somatic copy-number alteration across human cancers. *Nature* **463**, 899 (2010).
26 Network, C. G. A. R. Comprehensive molecular characterization of gastric adenocarcinoma. *Nature* **513**, 202 (2014).
27 Cristescu, R. *et al.* Molecular analysis of gastric cancer identifies subtypes associated with distinct clinical outcomes. *Nat. Med.* **21**, 449 (2015).
28 Cowley, G. S. *et al.* Parallel genome-scale loss of function screens in 216 cancer cell lines for the identification of context-specific genetic dependencies. *Sci. Data* **1**, 1-12 (2014).
